# Medication use and risk of proximal colon cancer: a systematic review of prospective studies with narrative synthesis and meta-analysis

**DOI:** 10.1007/s10552-021-01472-8

**Published:** 2021-07-05

**Authors:** Rhea Harewood, Ruth Disney, James Kinross, Christian von Wagner, Amanda J. Cross

**Affiliations:** 1 Department of Epidemiology and Biostatistics, School of Public Health, Imperial College London, London, UK; 2 Cancer Screening and Prevention Research Group (CSPRG), Department of Surgery and Cancer, Imperial College London, London, UK; 3 Department of Surgery and Cancer, Imperial College London, London, UK; 4 Research Department of Behavioural Science and Health, University College London, London, UK

**Keywords:** Medication, Proximal colon cancer, Risk factor, Etiology, Systematic review, Meta-analysis

## Abstract

**Purpose:**

Evidence of differences in the etiology of, and poorer survival from, proximal colon compared to the distal colorectum, necessitates research into its risk factors. This systematic review summarizes the evidence on medication use and proximal colon cancer risk.

**Methods:**

MEDLINE and EMBASE were searched for prospective studies investigating nine medication groups, namely non-steroidal anti-inflammatory drugs (NSAIDs), exogenous hormones, i.e., hormone replacement therapy (HRT) or oral contraceptives (OCs), statins, proton pump inhibitors, anti-hypertensives, metformin (an antidiabetic), antidiarrheals or laxatives, and the risk of proximal colon cancer. Narrative synthesis and meta-analyses, using random effects models to estimate risk ratios (RRs) and 95% confidence intervals (CIs), were conducted.

**Results:**

Twenty nine publications investigating NSAIDs (*n* = 13), exogenous hormones [HRT (*n* = 9) or OCs (*n* = 4)] statins (*n* = 5), anti-hypertensives (*n* = 1), and metformin (*n* = 1) were included. Summary RRs reported a protective effect of aspirin use (RR 0.80, 95% CI 0.73–0.89) but no associations between HRT (RR 0.92, 95% CI 0.83–1.02), OC (RR 1.06, 95% CI 0.98–1.14) or statin use (RR 0.94, 95% CI 0.67–1.31), and proximal colon cancer incidence compared to never/non-use. One study on metformin and one on anti-hypertensives reported no association. Sources of between-study heterogeneity included study design, period of exposure ascertainment, exposure source, and exposure comparison, but this exploration was hindered by the small numbers of studies.

**Conclusion:**

Despite some studies on NSAID or HRT use, evidence on the impact of a range of medications on proximal colon cancer risk is limited. This highlights the need for more research to inform chemoprevention strategies.

**Supplementary Information:**

The online version contains supplementary material available at 10.1007/s10552-021-01472-8.

## Introduction

Globally, colorectal cancer (CRC) is the fourth most common cancer and third leading cause of cancer death [1]. Screening programs have played an integral role in the reduction of CRC incidence and mortality [2]. However, despite this success, there is disparity in the detection of tumors by subsite of the colon [3, 4] with a disadvantage for proximal tumors due to their location further along the colon and often flat morphology.

The growing body of literature establishing the difference between proximal and distal tumors in genetic and molecular pathways of carcinogenesis, tumor progression, and survival [5, 6] supports the hypothesis of variation in risk factors by tumor location. Research focused solely on overall CRC risk factors may be overlooking significant associations for specific subsites. Therefore, studies into the etiology of proximal colon cancer are necessary to inform primary prevention strategies complementary to screening.

Knowledge of the mechanistic pathways through which cancer develops has led to increasing epidemiological research on the association between medications and cancer incidence. Most studies have focused on the protective effect of non-steroidal anti-inflammatory drugs (NSAIDs), including aspirin on CRC through, for example, inhibition of the cyclooxygenase (COX) enzymes [7, 8]. It is also plausible that some other widely used drugs have a protective effect. Anti-hypertensive drugs, especially angiotensin-converting enzyme (ACE) inhibitors or angiotensin II receptor blockers (ARBs), may reduce CRC incidence due to their inhibition of the hormone angiotensin II, which has been shown to promote cell proliferation, angiogenesis, inflammation, and metastasis [9]. Statins can inhibit the cell cycle and survival and induce apoptosis [10], and metformin (a first-line anti-diabetic drug) affects the insulin-like growth factor (IGF) pathway of CRC development among others [11]. Moreover, the increased incidence of proximal colon cancer in women and especially those post-menopause, highlights the potential role of exogenous estrogen and/or progesterone hormones in cancer reduction specifically in this subsite [12, 13]. Other drugs have a direct impact on the functioning of the colorectum. These include antidiarrheals [14] and laxatives, where fiber laxatives may have the potential to reduce CRC incidence and non-fiber laxatives to promote cancer development [15, 16]. There is also concern about the overuse of proton pump inhibitors (PPIs) [17, 18], as the resulting higher gastrin levels in the colorectum has been linked to carcinogenesis.

Despite several systematic reviews on this topic, most have focused on the association between one type of medication and CRC more generally [19–33]. To our knowledge, none have comprehensively examined a range of medications and explicitly focused on proximal colon cancer as the outcome. The reduced effectiveness of screening in this subsite highlights the importance of summarizing the evidence specific to proximal colon cancer to better understand the role of medications in its development and to inform complementary prevention or high-risk monitoring strategies.

The aim of this review was to provide a comprehensive summary and evaluation of the epidemiological evidence investigating common medications, including NSAIDs, exogenous hormones [hormone replacement therapy (HRT) or oral contraceptives (OC)], anti-hypertensives, statins, metformin, antidiarrheals, laxatives and PPIs, in relation to proximal colon cancer.

## Methods

This review was registered in the International Prospective Register of Systematic Reviews (PROSPERO, registration number: CRD42020172031). Findings from this review are reported according to Preferred Reporting Items for Systematic Reviews and Meta-Analyses (PRISMA) guidelines [34].

### Exposures of interest

The specific medications under review were NSAIDs, exogenous hormones (HRT or OC), anti-hypertensives (e.g., ACE inhibitors or ARBs), statins, metformin, antidiarrheals, laxatives and PPIs.

### Eligibility criteria

Randomized controlled trials (RCTs) or prospective observational studies investigating the association between medication use and proximal colon cancer incidence, in adults aged 18 or over were eligible for this review. Studies conducted in populations at a high risk for CRC, for example those with prevalent cancer or a history of CRC and those with Lynch syndrome or inflammatory bowel disease, were excluded. Studies where the outcome was incidence of proximal colon polyps, adenomas, or mortality, or survival from proximal colon cancer, were deemed ineligible. Case reports, case-studies, cross-sectional studies, non-nested case–control studies, editorials, commentaries, reviews, meta-analyses, and conference abstracts were also excluded.

### Search strategy

Two databases, MEDLINE and EMBASE, were searched from inception up to 30th April 2021 via OVID. Concepts were identified for the search relating to the exposures (medication), outcome (proximal colon cancer), study design (RCT, cohort study, nested case–control study), and effect measures. Keywords and Medical Subject Headings (MeSH) terms were identified for each concept and adapted for each database. For the concept relating to medication use, general “medication” or “drug” and specific drug MeSH terms were identified. The Scottish Intercollegiate Guidelines Network (SIGN) search filter for RCTs [35] and the British Medical Journal Best Practice study design search filters for cohort and nested case–control studies [36] were used to restrict on study design. No restriction or limits were defined based on time, geography, or language. Native speakers were consulted for the review of non-English articles. See Online Appendix Table 1 for search terms.

### Study selection

All journal articles identified were exported to EndNote and duplicates were removed based on automated pre-defined criteria and through manual screening. Screening of titles and abstracts was performed by one reviewer according to pre-defined inclusion and exclusion criteria; articles considered eligible were further examined by review of the full text by two reviewers independently and any discrepancies were resolved by a third party. The citations of the included studies were reviewed for additional eligible publications.

### Data extraction

Data from each study were extracted into standard tables highlighting information on: study design, study characteristics, participant characteristics, medication(s), exposure and outcome ascertainment, outcome definition, and effect measures. Measures of relative risk, expressed as a hazard ratio (HR), risk/rate ratio (RR) or odds ratio (OR) for nested case–control studies, with 95% confidence intervals (CIs), for the effect of specific medication use on the incidence of proximal colon cancer were extracted. Where more than one publication presented results from analyses on the same population and medication exposure, the study where estimates of risk were reported was extracted. Where this was the case for both publications, the one with the greater number of proximal colon cancer cases available for analysis and/or longer follow-up was extracted. The estimate presented in the final fully adjusted models was extracted where more than one effect estimate was reported.

### Risk of bias assessment

Observational studies were evaluated using the Newcastle–Ottawa Scale (NOS) for assessing the quality of nonrandomized studies in meta-analyses [37]. This assesses studies and assigns a score out of nine. In line with other systematic reviews [28, 38], included studies with a score of seven or above were designated as having an overall low risk of bias. For RCTs, the Cochrane Collaboration tool for assessing risk of bias in RCTs was used [39].

Studies were also evaluated for potential biases common in pharmacoepidemiological studies as informed by The European Network of Centres for Pharmacoepidemiology and Pharmacovigilance (ENCePP) Guide on Methodological Standards in Pharmacoepidemiology [40].

### Data synthesis

Where feasible, random effects meta-analyses were performed. Summary RRs (since proximal colon cancer is a rare outcome, ORs were also assumed to approximate risk) and 95% CIs were estimated using the DerSimonian and Laird method and presented along with individual study estimates in forest plots. Individual study estimates were log-transformed prior to generation of the pooled estimate. Heterogeneity between studies was examined using the *I*^2^ statistic and the associated p-value, which describes the proportion of variation within the pooled estimates due to between-study heterogeneity [41]; an *I*^2^ of above 50% was evidence of substantial heterogeneity [42, 43]. The risk of publication bias was assessed by visual inspection of funnel plots and using the Egger’s regression test. Subgroup and, where possible, meta-regression analyses, were conducted to assess biological and methodological sources of heterogeneity, i.e., sex, study design, source of exposure information (database, questionnaire, or RCT), period exposure information was ascertained and exposure comparisons (current use vs. never or non-use, ever vs. never use, or use vs. non-use).

Due to heterogeneity of the study designs, study populations and exposure definitions, a narrative synthesis informed by the Synthesis Without Meta-analysis (SWiM) guidelines was also used [44]. The certainty of the evidence presented for any given medication category was evaluated based on: the number of studies investigating the medication, the number of participants and events, the consistency of the effects across studies, directness of effect estimates to the research question, and the risk of bias.

Estimates for the broadest and most recent definition of use compared to never users or non-users were used in graphics (with priority given to current use) and the reporting of results to increase comparability of findings between included studies. Where an overall measure of ‘use’ was not presented (i.e., only an estimate stratified by dose or duration was presented), RRs or ORs and 95% CIs (using the exact method) were calculated based on the number of events for each exposure group and the numbers of participants or person-time reported in the manuscript. If these numbers were not available, the stratified estimates were pooled using random effects meta-analysis methods to obtain a summary estimate for medication use.

Analyses were conducted using Stata ® version 17.0 [45].

## Results

### Search results

**Fig. 1:**
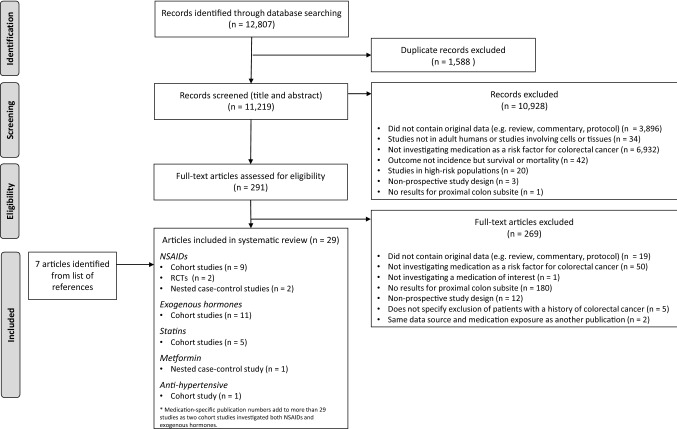
PRISMA flow chart of systematic review results. *RCTs* randomized controlled trials, *NSAIDs* non-steroidal anti-inflammatory drugs

The search strategy identified 12,807 publications for review, which included 1,588 duplicates (Fig. 1). After review of titles and abstracts, 291 remained for full-text assessment. An additional 269 articles were removed after full-text review, mostly due to the ineligibility of those not reporting results specifically for the proximal site of the colon (67%). Citation tracking of the remaining 22 publications identified an additional 7 leading to a total of 29 publications for inclusion.

### Study characteristics

The 29 included studies investigated associations between anti-hypertensives (one study) [46], exogenous hormones (HRT and OC) (11 studies) [47–57], metformin (one study) [58], NSAIDs (13 studies) [50, 56, 59–69], and statins (five studies) [70–74] (Fig. 1). However, no studies investigating PPIs, anti-diarrheals or laxatives were identified.

Included studies comprised 24 cohort studies, three nested case–control studies, two investigating NSAIDs, and one metformin, and two RCTs both investigating NSAIDs. All were conducted in high-income countries throughout Asia, North America, and Europe (Table 1).

**Table 1:** Overview of characteristics of the publications (*n* = 29) included in systematic review

Characteristic	Studies, *n* (%)^a^
Study	
Type	
Observational	
Cohort	24 (83)
Nested Case–Control	3 (10)
Randomized controlled trial	2 (7)
Region (Country)	
Asia	
Hong Kong	3 (10)
Korea	1 (3)
North America	
Canada	1 (3)
United States	18 (62)
Europe	
Denmark	1 (3)
France	1 (3)
United Kingdom	2 (7)
Sweden	1 (3)
Europe (10 countries)	1 (3)
Medication category^b^	
Anti-hypertensives	1 (3)
Exogenous hormones	
Hormone replacement therapy	9 (31)
Oral contraceptives	4 (14)
Metformin	1 (3)
NSAIDs	13 (45)
Statins	5 (17)
Exposure ascertainment	
Self- or interview-administered questionnaire	19 (66)
Prescription database	8 (28)
Not applicable (RCT)	2 (7)
Exposure definition	
Ever use	7 (24)
Use (defined period)	7 (24)
Current use	15 (52)
Exposure measurement	
Fixed	22 (76)
Time-varying	7 (24)
Outcome ascertainment	
Registry data	16 (55)
Self-report with medical record verification	13 (45)
Proximal colon cancer definition	
Caecum to hepatic flexure	1 (3)
Caecum to transverse colon^c^	10 (34)
Caecum to splenic flexure^c^	10 (34)
Not reported	8 (28)
Population	
Sample size	
< 10,000	1 (3)
10,000–99,999	18 (62)
≥ 100,000	10 (34)
Sex	
Women only^d^	15 (52)
Men only	1 (3)
Both	13 (45)
Follow-up time years (mean/median)	
< 5^e^	1 (3)
5–9	4 (14)
≥ 10^f^	18 (62)
Not applicable^g^	6 (21)

*NSAID* Non-steroidal anti-inflammatory drug, *RCT* Randomized controlled trial

^a^Some proportions do not add to 100% due to rounding

^b^Studies do not total 29 as more than one medication could be investigated within one study

^c^Some studies excluded the appendix

^d^Nine studies were investigating exogenous female hormones, therefore male participants were not applicable

^e^Mean nor median follow-up reported, but time from recruitment to end of follow-up was less than 5 years

^f^Mean nor median follow-up reported for two studies, but time from recruitment to end of follow-up was ≥ 10 years

^g^The outcome measure for three studies was post-colonoscopy proximal colon cancer within 3 years

In observational studies, medication use was commonly ascertained through self- or interview-administered questionnaires (66%), with the remainder utilizing prescription databases. The majority of studies reported on current use of medication at baseline or during a trial for RCTs (52%). Seven studies (24%) included exposure status as a time-varying variable in statistical models. Data on outcomes came from cancer or vital statistics registries (55%), or were self-reported and subsequently confirmed by review of medical records. For three studies [46, 67, 70] the outcome of interest was post-colonoscopy CRC within 3 years, with all other studies reporting on any CRC incidence as the outcome. Proximal colon cancer was mostly classified as the subsite from the caecum to the splenic flexure (34%); however, this information was not reported in eight of the studies. Approximately one-third of studies included ≥ 100,000 participants and in 18 studies (62%) participants were followed for at least 10 years. The studies are summarized in Online Appendix Tables 2–6.

### Risk of bias assessment

The risk of bias assessment is reported in Online Appendix Table 7. Of the 27 observational studies, 10 (37%) were assigned a score of less than seven. For cohort studies, the main sources of bias were the written self-report of exposure information (75%) and the use of convenience samples for the study population (29%). There was also a lack of reporting on the proportion of participants who were lost to follow-up (50%). The risk of bias for nested case–control studies was low for all domains. Risk of bias among RCTs was generally low; however, it was unclear whether the allocation sequence was concealed in either trial.

All observational studies used non-users or never users as comparators and used prevalent user designs, instead of incident user designs. The definition of cohort entry was the same for exposed and unexposed groups in most observational studies. However, one cohort study [73] used a prescription dataset to classify exposure and differentially defined cohort entry for statin users (date of prescription) compared to non-users (date of dyslipidaemia diagnosis or prescription of non-statin drug) and in another study definitions of cohort entry were unreported [52]. In the three included nested case–control studies, cases and controls were matched on index date (CRC diagnosis) [60] or the index date was defined as the date of baseline colonoscopy in both cases and controls [58, 68].

### NSAIDs

**Fig. 2:**
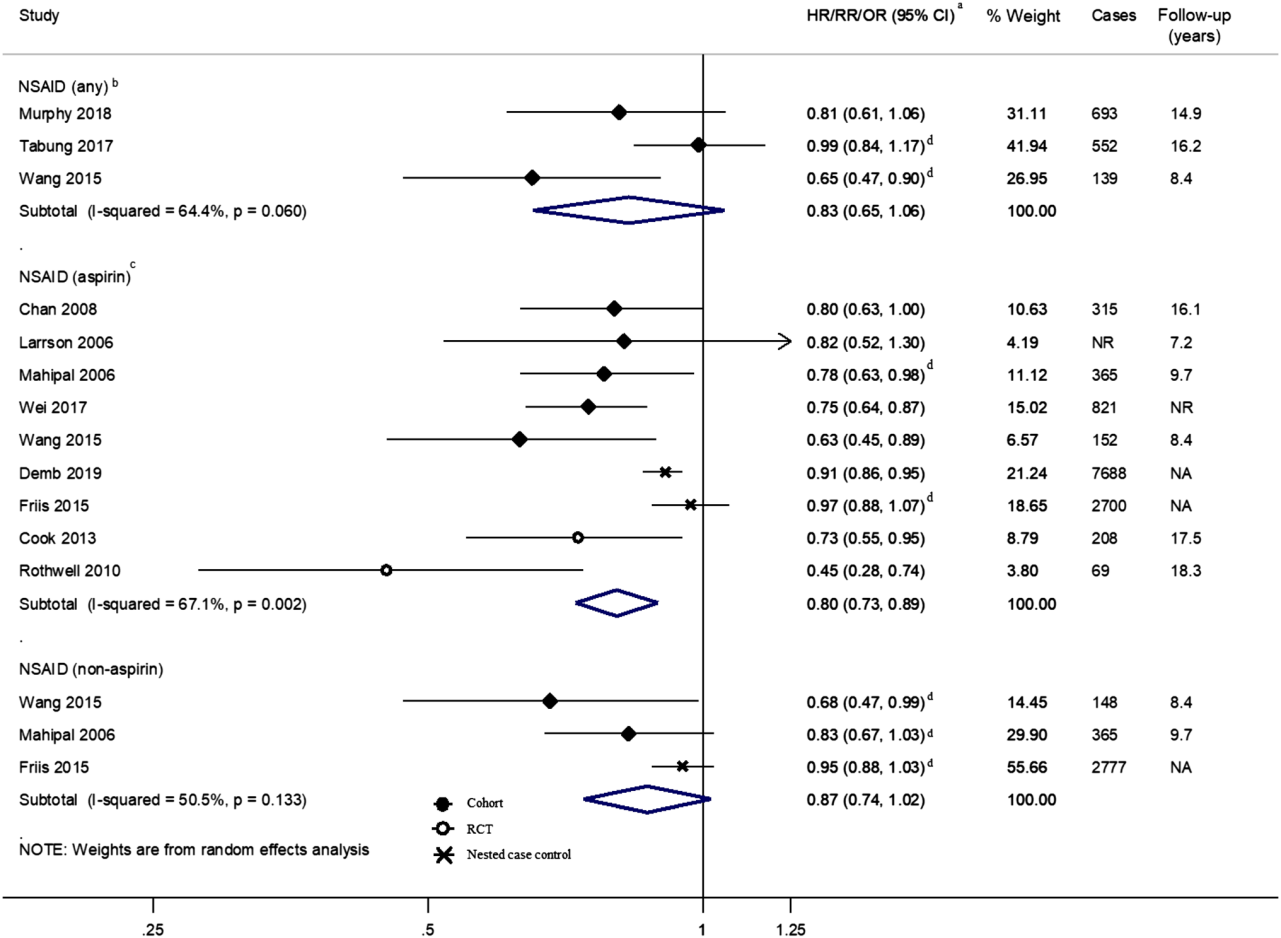
Risk estimates reported for the association between NSAID use and proximal colon cancer by medication type and study design. ^a^Estimates were log-transformed prior to generation of the pooled estimate; ^b^Excluding Cheung 2020 [67] due to different outcome measure, i.e., post-colonoscopy proximal colon cancer within 3 years; ^c^Excluding Allison 2006 [59] for which no association was found but no risk estimate was reported; ^d^Crude estimate reported as calculated from the number of events and the numbers of participants or person-time reported in article. *CI* Confidence interval, *HR* Hazard Ratio, *I-squared* test for heterogeneity, *NA* Not Applicable, *NR* Not reported, *NSAID* Non-steroidal anti-inflammatory drug, *OR* Odds Ratio, *p p*-value for heterogeneity, *RCT* Randomized controlled trial, *RR* Risk/Rate Ratio

Four studies [50, 63, 64, 67] examined the effect of any NSAID use on proximal colon cancer risk and found a general reduction in risk compared to non-users across studies (Fig. 2). This reduction was statistically significant in Wang et al. [64] (HR 0.65; 95% CI 0.47–0.90; univariate estimate calculated from the number of participants and events) but the number of events were small comparatively (*n* = 139). Cheung et al. [67] also reported an inverse association for aspirin use (RR 0.48; 95% CI 0.24–0.95) but the outcome was proximal colon cancer within 3 years of index colonoscopy which could be reflective of the impact of aspirin on progression of lesions missed during colonoscopy, affecting its comparability to the other studies included in the review and so was not included in the figure.

Nine studies [56, 59–62, 65, 66, 68, 69] focused on aspirin specifically. Six studies reported a statistically significant protective effect for aspirin use compared to non-use. In the two RCTs, Cook et al. [69] reported a significantly decreased risk, HR 0.73; 95% CI 0.55–0.95 in the intervention group (aspirin on alternate days), after an extended follow-up of 17 years and Rothwell et al. [62], with a median follow-up of 18.3 years, noted significant reductions in proximal colon cancer risk, HR 0.45; 95% CI 0.28–0.74 in the intervention group (daily aspirin compared to placebo). The largest cohort study [56] investigated aspirin use in the Nurses’ Health Study (NHS) cohort and found a decreased risk of proximal colon cancer in women using seven tablets per week per year (*n* = 821 cases, HR 0.75; 95% CI 0.64–0.87). Demb et al. [68] reported data from a nested case–control study with the largest number of accumulated proximal colon cancer cases (*n* = 7,688) finding a statistically significant decreased risk with aspirin use (≥ 2 prescriptions/mentions in electronic health record database) compared to non-use; however, another large nested case–control study [60] (*n* = 2,700 cases) reported no association. Of the three studies investigating non-aspirin NSAIDs and proximal colon cancer, only one found a statistically significant inverse association with proximal colon cancer [64] (Fig. 2).

**Fig. 3:**
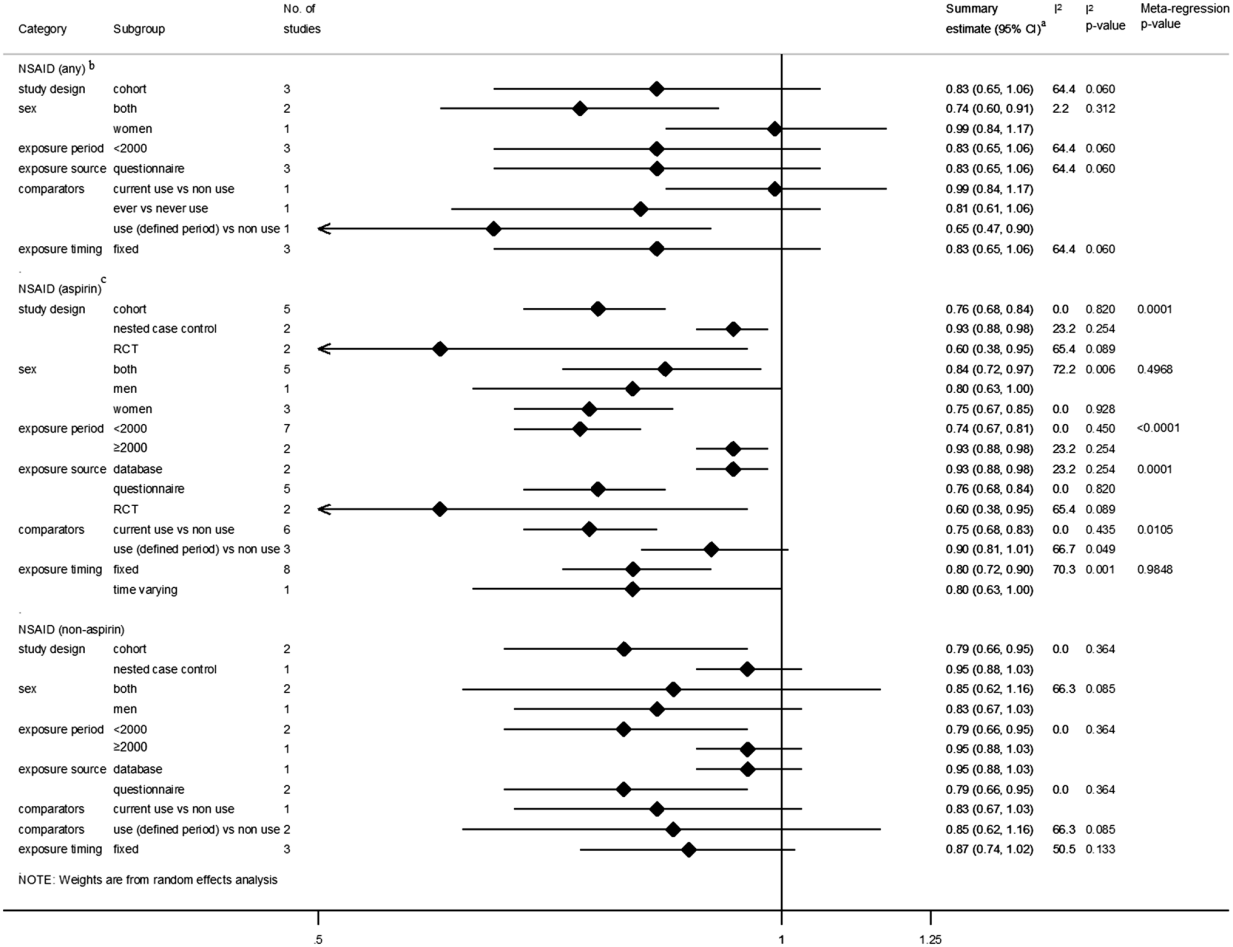
Summary risk estimates reported for the association between NSAID use and proximal colon cancer by subgroup. ^a^Estimates were log-transformed prior to generation of the pooled estimate; ^b^Excluding Cheung 2020 [67] due to different outcome measure, i.e., post-colonoscopy proximal colon cancer within 3 years; ^c^Excluding Allison 2006 [59] for which no association was found but no risk estimate was reported. *CI* Confidence interval, *I*^*2*^ test for heterogeneity between studies within subgroups, *Meta-regression p-value p*-value for evidence of between subgroup heterogeneity, *NSAID* Non-steroidal anti-inflammatory drug

For aspirin use, the pooled summary estimate revealed a statistically significant decreased risk of proximal colon cancer but there was strong evidence of between-study heterogeneity (*I*^2^ = 67.1%, *p* = 0.002). Pooled estimates for any NSAID use and non-aspirin NSAID use were not statistically significant, with borderline evidence of heterogeneity between studies for any NSAID use (*I*^2^ = 64.4%, *p* = 0.060). This heterogeneity was explored in subgroup analyses (Fig. 3). For studies investigating aspirin use, all but three strata indicated statistically significant decreases in proximal colon cancer risk. Meta-regression revealed study design (*p* = 0.0001), year of exposure status ascertainment (*p* < 0.0001), source of exposure status information (*p* = 0.0001), and comparator definitions as sources of heterogeneity (*p* = 0.0105) (Fig. 3).

Subsite heterogeneity was also explored within included studies. Where results from a statistical test were reported, one RCT [62] found a decreased risk of proximal colon cancer not observed for distal colon cancer. This was also observed for NSAID and aspirin use in one large cohort study [64]  after pooling of stratified estimates. In the other studies, no differences [50, 56, 61] or reduced risks for both proximal colon and distal colon cancer were reported [68].

Funnel plots and the Egger’s test found evidence of publication bias for studies investigating aspirin (*p* = 0.011) and non-aspirin NSAIDs (*p* = 0.045).

### Exogenous hormones (HRT and OCs)

**Fig. 4:**
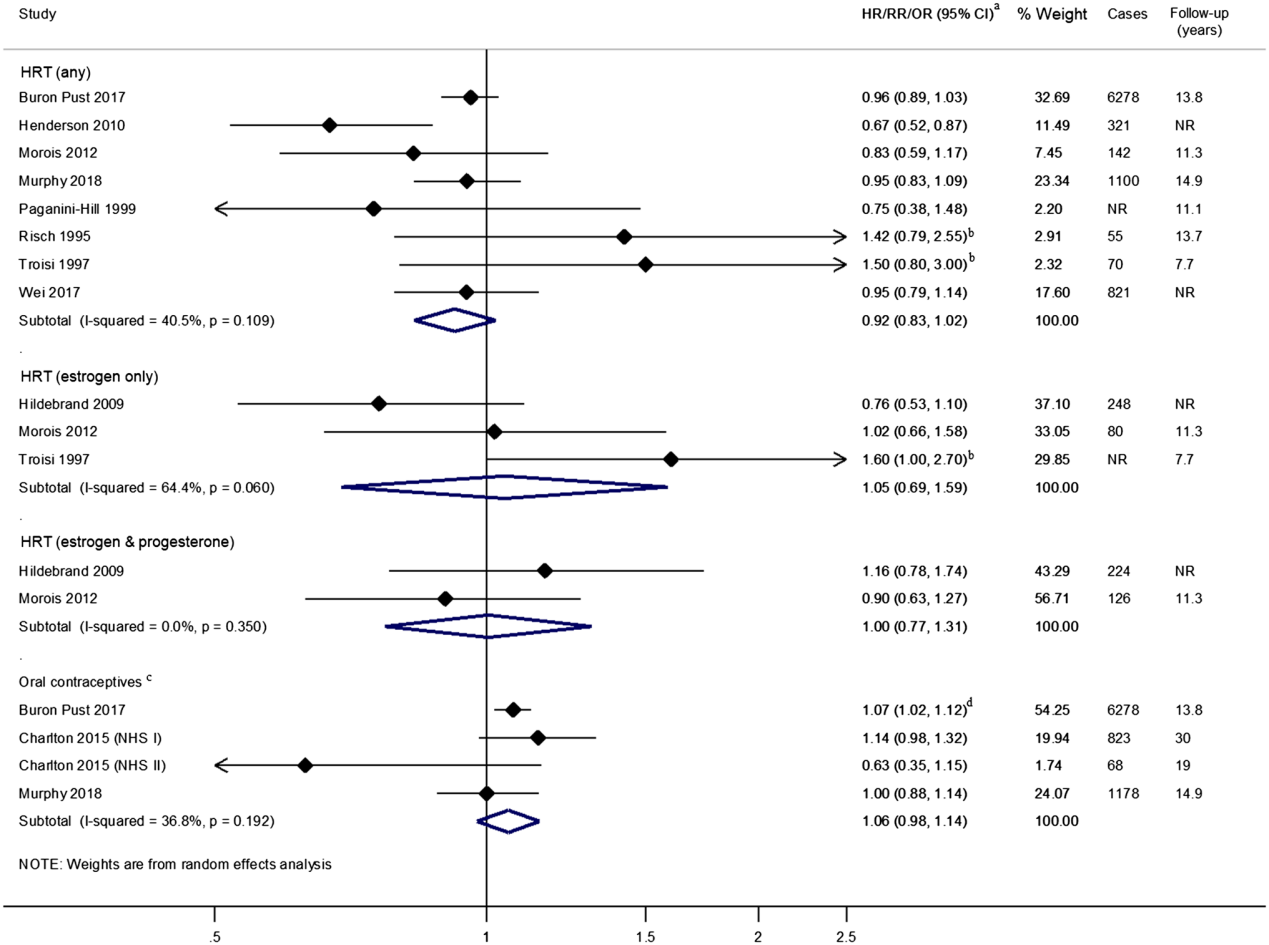
Risk estimates reported for the association between exogenous hormone use and proximal colon cancer by medication type. ^a^Estimates were log-transformed prior to generation of the pooled estimate; ^b^Estimate only age-adjusted; ^c^Excluding Lin 2007 [54] for which no association was found but no risk estimate was reported; ^d^Summary estimate obtained from stratified estimates using random effects meta-analysis methods. *CI* Confidence interval, *HR* Hazard Ratio, *HRT* Hormone Replacement Therapy, *I-squared* test for heterogeneity, *OR* Odds Ratio, *NR* Not reported, *p p*-value for heterogeneity, *R* Risk/Rate Ratio

Eight studies [47, 49–53, 55, 56] focused on any HRT use; all but one [52] studied its use in post-menopausal women. In most studies, even though the point estimates indicated a decreased risk, only Henderson et al. [55] found a significant inverse association with proximal colon cancer incidence based on 321 cases (Fig. 4). The two studies using data from the Million Women’s study [47] and the European Prospective Investigation into Cancer and Nutrition cohort (~ 500,000 participants) [50] for which estimates were based on over 1,000 proximal colon cases and with ~ 14 years of follow-up, reported no association. In pooled analyses, no association between HRT use and proximal colon cancer incidence was observed compared to never or non-use, summary RR 0.92; 95% CI 0.83–1.02.

The summary estimate from three studies [48, 49, 53] found no evidence of an association of estrogen-only HRT use compared to never or non-use with proximal colon cancer incidence (RR 1.05; 95% CI 0.69–1.59). In the two studies [48, 49] of women using a combination of estrogen and progesterone HRT, the findings were also null, summary RR 1.00; 95% CI 0.77–1.31.

Four studies [47, 50, 54, 57] examined the risk of proximal colon cancer in women taking OCs (ever users compared to never users) and all followed participants for over 10 years. Lin et al. [54] reported no association with OC use; however, the specific risk estimate was not reported. Pooled analysis of the remaining three studies [47, 50, 57], one of which included estimates from two cohorts NHS I and II [57], also found no association between OC use and proximal colon cancer incidence, summary RR 1.06; 95% CI 0.98–1.14. Only one study [47] included in the pooled analysis found a significant increased risk, RR 1.07; 95% CI 1.02–1.12 [pooled estimate based on RRs stratified by duration of OC use (< 5 years and ≥ 5 years)].

**Fig. 5:**
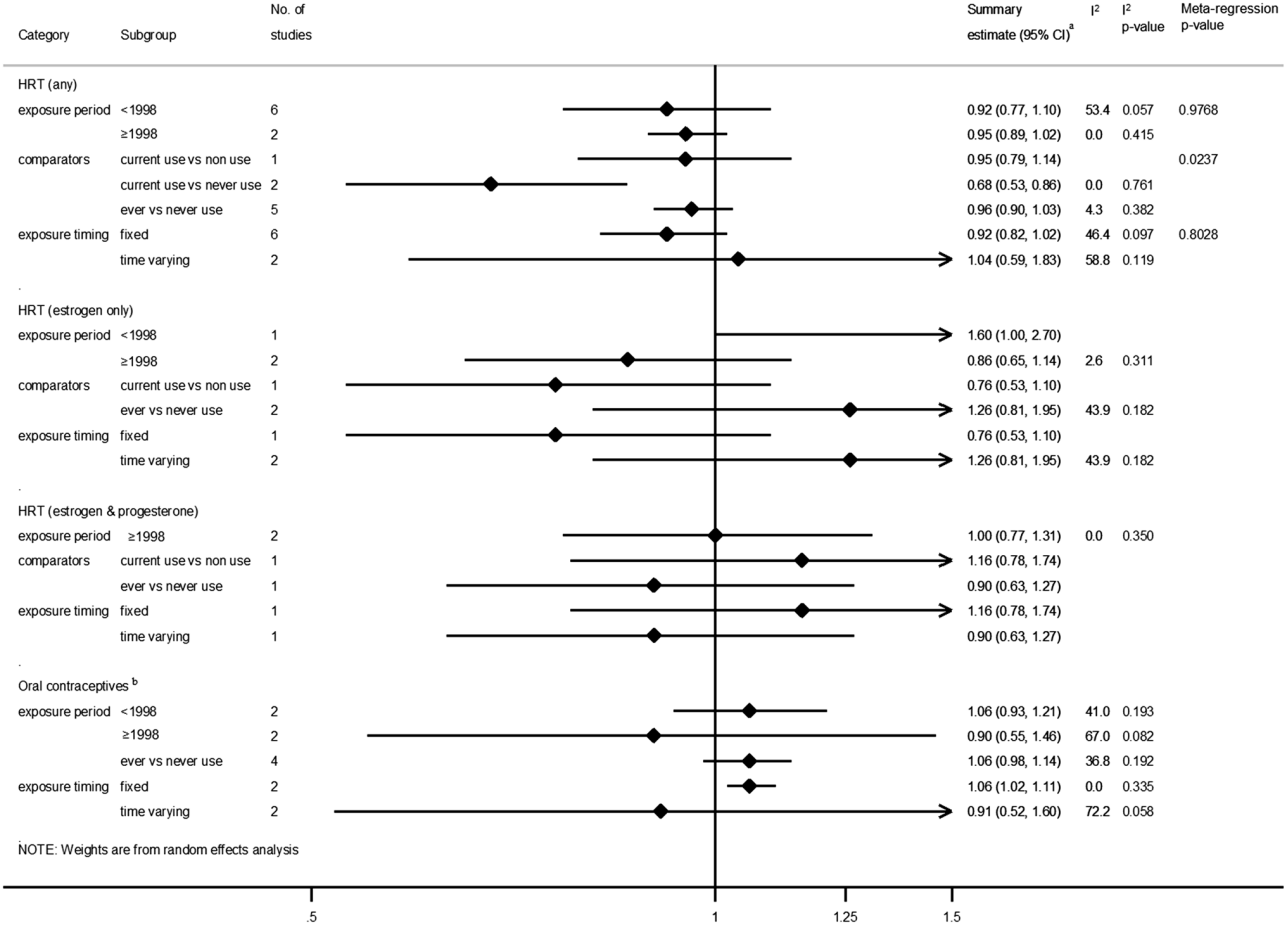
Summary risk estimates reported for the association between hormone use and proximal colon cancer by subgroup. ^a^Estimates were log-transformed prior to generation of the pooled estimate; ^b^Excluding Lin 2007 [54] for which an association but no specific risk estimate was reported. *CI* Confidence interval, *HRT* Hormone Replacement Therapy, *HR* Hazard Ratio, *I*^*2*^ test for heterogeneity between studies within subgroups, *Meta-regression p-value p*-value for evidence of between subgroup heterogeneity

Heterogeneity between studies was assessed by year(s) of exposure use ascertainment [using a cut-off of 1998 when HRT use decreased following evidence of adverse side effects [75]] and definitions of exposure use. In subgroup analysis, only studies investigating current use vs. never use among studies investigating any HRT use or those which treated exposure status as a fixed quantity in statistical models among those investigating OCs showed a statistically significant decreased risk in proximal colon cancer (Fig. 5). Meta-regression analyses were only feasible for studies involving any HRT use. Comparator definition was shown to be a source of heterogeneity (*p* = 0.0237) but small numbers of studies between subgroups may have affected this result (Fig. 5).

Of studies investigating HRT or OC use, only three reported tests for heterogeneity in estimates for proximal compared to distal colon cancer and only one [49] indicated a difference in effect and direction (decreased risk for distal colon cancer compared to no association for proximal colon cancer) for estrogen-only HRT use.

Funnel plots and the Egger’s test did not detect evidence of publication bias for studies investigating HRT or OC use (data not shown).

### Statins

**Fig. 6:**
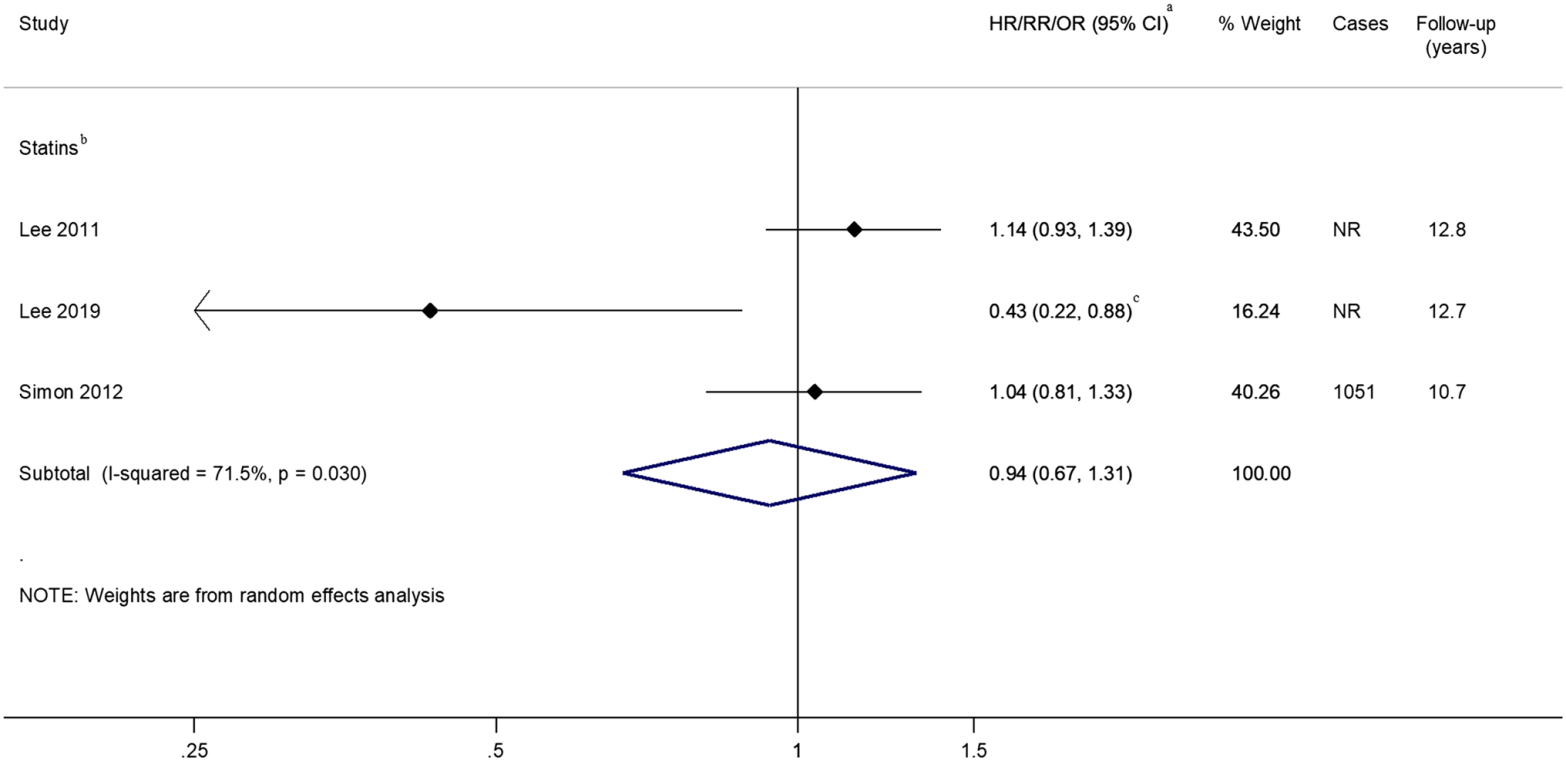
Risk estimates reported for the association between statin use and proximal colon cancer. ^a^Estimates were log-transformed prior to generation of the pooled estimate; ^b^Excluding Cheung 2019 [70] due to different outcome measure, i.e., post-colonoscopy proximal colon cancer within 3 years and Jacobs 2006 [71] for which an association but no specific risk estimate was reported; ^c^Summary estimate obtained from stratified estimates using random effects meta-analysis methods. *CI* Confidence interval, *HR* Hazard Ratio, *I-squared* test for heterogeneity, *OR* Odds Ratio, *NR* Not reported, *p p*-value for heterogeneity, *RR* Risk/Rate Ratio

Five studies [70–74] investigated the use of statins (or other cholesterol-lowering drugs) and proximal colon cancer incidence, with three reporting follow-up of over 10 years [72–74]. Jacobs et al. [71] did not present subsite-specific RRs but reported that there was no association between statin use vs. non-use and proximal colon cancer, while two [70, 73] of the remaining four studies reported significant inverse associations. Pooled analyses including three studies [excluding Cheung 2020 [70] where the outcome was post-colonoscopy proximal colon cancer within 3 years and therefore was not comparable with the other studies] revealed no association between statin use and proximal colon cancer incidence, summary RR 0.94; 95% CI 0.67–1.31 (Fig. 6); although there was significant heterogeneity between studies (*I*^2^ = 71.5%, *p* = 0.030).

**Fig. 7:**
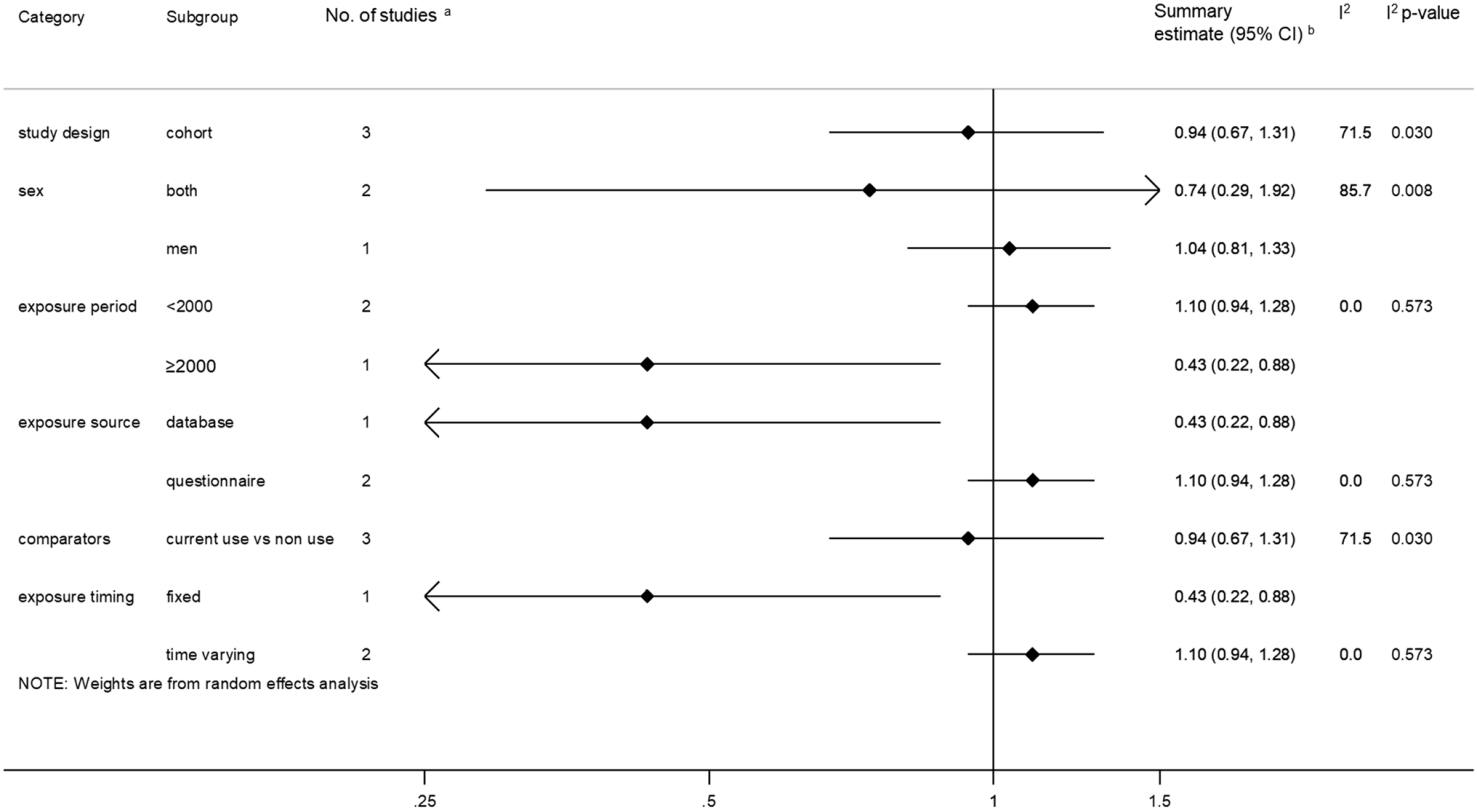
Summary risk estimate reported for the association between statin use and proximal colon cancer by subgroup. ^a^Excluding Cheung 2019 [70] due to different outcome measure, i.e., post-colonoscopy proximal colon cancer within 3 years and Jacobs 2006 [71] for which no association was found but no specific risk estimate was reported; ^b^Estimates were log-transformed prior to generation of the pooled estimate. *CI* Confidence interval, *I*^*2*^ test for heterogeneity between studies within subgroups, *Meta-regression p-value p*-value for evidence of between subgroup heterogeneity

Subgroup analyses were conducted, but, due to the small number of studies, it was not possible to conduct meta-regression analyses. Studies where exposure information was obtained in or after 2000, those where a database was used to retrieve exposure information or where the exposure status was used as a fixed quantity in statistical models were shown to be associated with a decreased risk of proximal colon cancer but these strata only included one study each (Fig. 7).

There was evidence of publication bias for studies investigating statin use, *p* = 0.002.

### Metformin

Only one nested case–control study [58] investigated the association between metformin use and proximal colon cancer incidence in diabetic patients and found no association, OR 0.96; 95% CI 0.88–1.04 (*n* = 2,625 cases) between metformin use for at least one year before baseline compared to non-use.

### Anti-hypertensives

One retrospective cohort study [46] examined the association between antihypertensive use [ACE inhibitors or ARBs] for at least 180 days within 5 years prior to index colonoscopy and post-colonoscopy proximal colon cancer diagnosed within 3 years of index colonoscopy. The study reported no association in users compared to non-users (HR 0.83; 95% CI 0.51–1.35).

## Discussion

To our knowledge, this is the first systematic review to focus on a range of medications and their association with proximal colon cancer, for which screening methods have been less effective compared to other CRC subsites. Included studies suggest a protective effect of NSAID use on proximal colon cancer risk, driven by observational studies and RCTs investigating aspirin use. However, included studies were highly heterogeneous. Conversely, there was limited or inconsistent evidence available to support an association between use of HRT, OC, statins, anti-hypertensives, or metformin compared to never or non-users and proximal colon cancer. Most included studies did not report results of statistical tests for heterogeneity in the effect of medication use on risk of proximal compared to distal colon cancer, with only two studies [62, 64] reporting a decreased risk of proximal colon cancer not observed for distal colon cancer among NSAID users compared to non-users. No studies were identified for antidiarrheals, laxatives or PPIs, reflecting the paucity of epidemiological literature on the topic.

A previous review by Tomic et al. [28] reported an inverse association between non-aspirin NSAID use and proximal colon cancer with a pooled OR of 0.73, 95% CI 0.60–0.87 based on five studies. Additionally, one meta-analysis focused on aspirin [76] noted a decrease in risk in CRC which did not differ by subsite in secondary analyses, but as the review focused on overall CRC as an outcome, these specific results were not shown.

Previous research linking chronic inflammation to CRC has led scientists to postulate a possible preventative role of NSAIDs. The most researched pathway is via the inhibition of the COX-2 enzyme. This enzyme is found in high concentrations in a large proportion of cancer cells and is involved in prostaglandin synthesis [77–79]. In cancer cells, prostaglandins are known to increase cell proliferation, inhibit cell death, and promote vascular invasion and metastasis [78]. Alternative pathways which promote apoptosis or the reduction of pro-carcinogenic polyamines [12], include the inhibition of nuclear factor kappa B (NF-κB) or activation of p38 kinase.

Contrary to this review, one by Grodstein et al. [80] reported an inverse association between HRT use and proximal colon cancer. Estrogens may play a more important role in the modulation of proximal colonic carcinogenesis specifically, with evidence of higher proportions of proximal colon cancer in women compared to men, and increasing with age in women [13]. Estrogen receptors (ER), specifically ERβ, have been identified along the colon epithelium and biological studies report a loss of this receptor in colonic carcinoma. This loss is differential depending on tumor location, with a greater reduction in the proximal colon [13, 81]. These receptors have also been associated with the incidence of microsatellite instability high tumors, which are more likely to be found in the proximal colon and in older women [13, 82].

A systematic review of observational studies by Liu et al. [24] reported a reduction for rectal but not colon cancer for statin users compared to non-users. Even though cell and animal studies lend support to the role of statins in the prevention of CRC, epidemiological studies have been less convincing. One proposed mechanism includes a reduction of phosphate by-products of the mevalonate pathway shown to be involved in tumor growth, angiogenesis, and metastasis [83].

Metformin is thought to have anti-tumor activity, such as reducing insulin resistance and hyperinsulinemia, which are associated with tumor growth [38], and inducing apoptosis [38]. Even though several meta-analyses have reported protective effects of metformin use on overall CRC risk [38, 84–87], only one study on metformin and proximal colon cancer was found in this review, and this reported no association [58]. Similarly, one systematic review investigated the association between ACE inhibitors and ARBs, which have the potential to promote tumor growth [88, 89], and found a 6% decreased risk in all-site CRC in pooled analysis [90]. However, yet again this review did not include sub-analyses by subsite of the colorectum.

Some limitations need to be considered for this review. Publications in which findings by subsite of the colon were only referred to in the body of the manuscript, and not within the abstract or title, would not have been identified using our search strategy. We reviewed citations in included studies to minimize the likelihood of this. There was evidence of considerable heterogeneity especially for studies investigating aspirin and statin use. Sources of heterogeneity for aspirin are mainly due to differences in study design, year, and source of exposure ascertainment, or comparator definition but the small numbers of studies for other medications precluded an in-depth statistical analysis of between-study heterogeneity.

Some studies utilized data from cohorts using convenience samples or specialized groups (e.g., nurses or other health professionals) to increase participant numbers and to decrease the probability of attrition. Such groups may have been more educated or health-conscious, which would have allowed for greater accuracy of the reporting of medication use; however, they would not have been representative of the general population. Medication use was most commonly reported using self-reported questionnaires, which are prone to recall bias and potential misclassification of the exposure, particularly for medications such as NSAIDs, as these drugs are often used for one-off or short-term treatments; however, since the outcome was ascertained after the exposure, this misclassification is likely to have occurred similarly between both cases and non-cases. In cohort studies, a lack of follow-up could have biased results if patient characteristics related to the outcome, for example age or socio-economic status, were different among those lost. The lack of reporting on follow-up in several studies made it difficult to evaluate any potential bias. Residual confounding may also have influenced results in studies where only age-adjusted estimates were presented.

Prevalent user designs were used in all observational studies included. This can lead to selection bias when only survivors of the early stage of medication use are included and is especially likely where exposure risk varies over time with higher risk in the short-term. For aspirin use, RCTs which used new user designs reported reduced risks of proximal colon cancer, however. Studies were also prone to surveillance bias where specific medication users are more likely to have proximal colon cancer detected due to more contact with health services compared to non- or never-users of medications; this may lead to an overestimation of risk in medication users and could have been addressed with the use of ‘active’ comparator groups but this was not employed in any of the studies. Immortal time bias could have resulted in an underestimation of risk and would have been likely when exposure status was obtained from prescription databases and follow-up time started for exposed participants at the date of the prescription with a previous start date for non-users of the medication under study. Most included studies using prescription databases cohort entry was the same for both exposed and unexposed and seven studies using self-reported medication use minimized this bias at the analysis stage by including exposure status as a time-varying variable. Nested case–control studies may be affected by time-window bias where there is a longer window to measure exposure in controls than in cases. The three nested case–control studies included in this review minimized the likelihood of this bias by either matching cases and controls on the index date or by defining the same time window for identification of exposures for both cases and controls. Protopathic bias is also a cause for concern for some studies where the start of the exposure medication is in response to symptoms from the outcome, thus potentially overestimating the effect of a medication. In most studies included in this analysis there was no time lag used between exposure status and diagnosis of proximal colon cancer thus increasing the likelihood of this bias for medications such as NSAIDs, which are used for pain relief.

This review has several strengths. Two of the largest bibliographic databases for medical journals were searched, and this was complemented by screening references lists of the eligible studies. The sensitivity of the searches was maximized by using drug class, brand-specific, or generic drug names as search terms to define the exposures of interest and no restrictions on language, place of publication, or study quality were defined. Only studies with a prospective study design were included to minimize the probability of information bias in exposure assessment or reverse causality, which are established issues in retrospective and cross-sectional studies; however, this resulted in fewer studies in our review.

In summary, there appears to be a suggestive inverse association between NSAID use and proximal colon cancer. However, there is limited consistent evidence or a lack of evidence to make definitive conclusions with respect to exogenous hormones, anti-hypertensives, statins, and metformin. Moreover, there is a scarcity of studies investigating the association between a range of medications and CRC risk by subsite, mostly due to the lack of statistical power to detect differences at this level of granularity. Studies of the etiology of proximal colon cancer are essential, especially considering that the later detection of cancers in the proximal colon compared to distal cancers results in advanced stage at diagnosis and lower survival. Our review has highlighted the need for more large, well-powered, high-quality research focused on different groups of medications, especially those for which in vitro and animal studies have reported an effect on tumor cells, to inform prevention strategies to complement screening.

## Supplementary Information

Below is the link to the electronic supplementary material.

Supplementary file1 (DOCX 133 kb)
