# Supplementary material for: Medication use and risk of proximal colon cancer: a systematic review of prospective studies with narrative synthesis and meta-analysis
Source: Cancer Causes Control. 2021 Jul 5;32(10):1047–61. doi: 10.1007/s10552-021-01472-8 (PMC8417019; doi:10.1007/s10552-021-01472-8)
Supplement: Supplementary file 1 — Supplementary file1 (DOCX 133 kb) [file 10552_2021_1472_MOESM1_ESM.docx]

**SUPPLEMENTARY APPENDIX**

**Cancer Causes & Control**

**Medication use and risk of proximal colon cancer: a systematic review of prospective studies with narrative synthesis and meta-analysis**

Rhea Harewood^1,2^, Ruth Disney^1,2^, James Kinross^3^, Christian von Wagner^4^, Amanda J Cross^1,2^

^1^ Department of Epidemiology and Biostatistics, School of Public Health, Imperial College London, London, UK

^2^ Cancer Screening and Prevention Research Group (CSPRG), Department of Surgery and Cancer, Imperial College London, London, UK

^3^ Department of Surgery and Cancer, Imperial College London, London, UK

^4^ Research Department of Behavioural Science and Health, University College London, London, UK

**Corresponding author:** Rhea Harewood, Department of Epidemiology and Biostatistics, School of Public Health, Imperial College London, St Mary’s Campus, Norfolk Place, London, W2 1PG, Email: [r.harewood18@imperial.ac.uk](mailto:r.harewood18@imperial.ac.uk), Tel: +44 20 7594 3272

**Appendix table 1:** Search strategies for each database included in the systematic review

| **EMBASE** | | |
| --- | --- | --- |
| 1 | | exp colorectal tumor/ or exp colon tumor/ or exp rectum tumor/ or exp cecum tumor/ |
| 2 | | ((Colorectal or colon or colonic or rectum or rectal or bowel or large intestin* or gut) adj3 (cancer* or carcinoma* or neoplasm* or neoplasia or tumo?r* or malignancy or adenoma* or adenocarcinoma or polyp or polyps)).mp. [mp=title, abstract, heading word, drug trade name, original title, device manufacturer, drug manufacturer, device trade name, keyword, floating subheading word, candidate term word] |
| 3 | | 1 or 2 |
| 4 | | (proximal or distal or site? or type? or subsite? or rightside? or right side? or leftside? or left side? or descending or ascending).mp. [mp=title, abstract, heading word, drug trade name, original title, device manufacturer, drug manufacturer, device trade name, keyword, floating subheading word, candidate term word] |
| 5 | | 3 and 4 |
| 6 | | exp antiinflammatory agent/ or exp antilipemic agent/ or exp acetylsalicylic acid/ or exp proton pump inhibitor/ or exp antihypertensive agent/ or exp laxative/ or exp antidiarrheal agent/ or exp hormone substitution/ or exp metformin/ |
| 7 | | (medication* or medicine* or drug* or statin* or aspirin* or warfarin or heparin or nonsteroidal or non-steroidal or antiinflammatory or anti-inflammatory or NSAID or NSAIDs or cyclooxygenase inhibitor or proton pump inhibitor* or proton-pump inhibitor* or antihypertensive* or anti-hypertensive* or laxative* or steroid or Emozul or Nexium or Losec or Mepradec or pantoprazole or loperamide hydrochloride or Imodium or amlodipine or diltiazem hydrochloride or Adizem or Dilzem or Slozem or Tildiem or felodipine or Cardioplen or Vascalpha or isosorbide or Chemydur or Monomax or lacidipine or lercanidipine or nifedipine or Adalat or Coracten or nicorandil or verapamil hydrochloride or indoramin or doxazosin or atenolol or bisoprolol or bisoprolol fumarate or carvedilol or atenolol or CoTenidone or Co-Tenidone or netoprolol or Betaloc or Lopresor or nebivolol or propranolol or sotalol or candesartan or Amias or eprosartan or irbesartan or losartan or olmesartan medoxomil or Olmetec or valsartan or arginine or perindopril or perindopril erbumine or Coversyl or captopril or enalapril or lisinopril or quinapril or ramipril or moxonidine or bendroflumethiazide or coamilozide or co-amilozide or hydrochlorothiazide or indapamide or Natrilix or furosemide or frusemide or Lasix or Lipitor or Simvador or bezafibrate or Bezalip or ezetimibe or Ezetrol or fenofibrate or Supralip or Clonidine or orlistat or loperamide or adalat or amlodipine or bendrofluazide or bisoprolol or candesartan cilexetil or diltiazem or enalapril or metoprolol or perindopril or diclofenac or ibuprofen or meloxicam or naproxen or cerazette or elleste duet or kliovance or premarin or fybogel orange or lactulose product or movicol oral powder or senna or aspirin or ????????statin or ??????????prazole or metformin).mp. [mp=title, abstract, heading word, drug trade name, original title, device manufacturer, drug manufacturer, device trade name, keyword, floating subheading word, candidate term word] |
| 8 | | 6 or 7 |
| 9 | | exp risk factor/ or exp incidence/ |
| 10 | | (Association* or effect or effects or incidence or risk* or incident* or rate ratio* or hazard* or odds ratio*).mp. [mp=title, abstract, heading word, drug trade name, original title, device manufacturer, drug manufacturer, device trade name, keyword, floating subheading word, candidate term word] |
| 11 | | 9 or 10 |
| 12 | | 5 and 8 and 11 |
| 13 | | Clinical Trial/ or randomized controlled trial/ or controlled clinical trial/ or multicenter study/ or Phase 3 clinical trial/ or Phase 4 clinical trial/ or exp RANDOMIZATION/ or Single Blind Procedure/ or Double Blind Procedure/ or Crossover Procedure/ or PLACEBO/ or Prospective Study/ |
| 14 | | randomi?ed controlled trial$.tw. or rct.tw. or (random$ adj2 allocat$).tw. or single blind$.tw. or double blind$.tw. or ((treble or triple) adj blind$).tw. or placebo$.tw. |
| 15 | | 13 or 14 |
| 16 | | Case Study/ or abstract report/ or letter/ |
| 17 | | case report.tw. or Conference proceeding.pt. or Conference abstract.pt. or Editorial.pt. or Letter.pt. or Note.pt. |
| 18 | | 16 or 17 |
| 19 | | 15 not 18 |
| 20 | | exp cohort analysis/ or exp longitudinal study/ or exp prospective study/ or exp follow up/ or exp case control study/ |
| 21 | | cohort$.tw. or (case$ and control$).tw. |
| 22 | | 20 or 21 |
| 23 | | exp cross-sectional study/ |
| 24 | | 22 not 23 |
| 25 | | 19 or 24 |
| 26 | | 25 and 12 |
| 27 | | exp animals/ or exp invertebrate/ or animal experiment/ or animal model/ or animal tissue/ or animal cell/ |
| 28 | | exp human/ or exp "human tissue, cells or cell components"/ |
| 29 | | 27 and 28 |
| 30 | | 27 not 29 |
| 31 | | 26 not 30 |
| **MEDLINE** | | |
| 1 | exp Colorectal Neoplasms/ | |
| 2 | ((Colorectal or colon or colonic or rectum or rectal or bowel or large intestin* or gut) adj3 (cancer* or carcinoma* or neoplasm* or neoplasia or tumo?r* or malignancy or adenoma* or adenocarcinoma or polyp or polyps)).mp. [mp=title, abstract, original title, name of substance word, subject heading word, floating sub-heading word, keyword heading word, organism supplementary concept word, protocol supplementary concept word, rare disease supplementary concept word, unique identifier, synonyms] | |
| 3 | 1 or 2 | |
| 4 | (proximal or distal or site? or type? or subsite? or rightside? or right side? or leftside? or left side? or descending or ascending).mp. [mp=title, abstract, original title, name of substance word, subject heading word, floating sub-heading word, keyword heading word, organism supplementary concept word, protocol supplementary concept word, rare disease supplementary concept word, unique identifier, synonyms] | |
| 5 | 3 and 4 | |
| 6 | exp Anti-Inflammatory Agents/ or exp Anticholesteremic Agents/ or exp ASPIRIN/ or exp Proton Pump Inhibitors/ or exp Antihypertensive Agents/ or exp LAXATIVES/ or exp Antidiarrheals/ or exp Hormone Replacement Therapy/ or exp metformin/ | |
| 7 | (medication* or medicine* or drug* or statin* or aspirin* or warfarin or heparin or nonsteroidal or non-steroidal or antiinflammatory or anti-inflammatory or NSAID or NSAIDs or cyclooxygenase inhibitor or proton pump inhibitor* or proton-pump inhibitor* or antihypertensive* or anti-hypertensive* or laxative* or steroid or Emozul or Nexium or Losec or Mepradec or pantoprazole or loperamide hydrochloride or Imodium or amlodipine or diltiazem hydrochloride or Adizem or Dilzem or Slozem or Tildiem or felodipine or Cardioplen or Vascalpha or isosorbide or Chemydur or Monomax or lacidipine or lercanidipine or nifedipine or Adalat or Coracten or nicorandil or verapamil hydrochloride or indoramin or doxazosin or atenolol or bisoprolol or bisoprolol fumarate or carvedilol or atenolol or CoTenidone or Co-Tenidone or netoprolol or Betaloc or Lopresor or nebivolol or propranolol or sotalol or candesartan or Amias or eprosartan or irbesartan or losartan or olmesartan medoxomil or Olmetec or valsartan or arginine or perindopril or perindopril erbumine or Coversyl or captopril or enalapril or lisinopril or quinapril or ramipril or moxonidine or bendroflumethiazide or coamilozide or co-amilozide or hydrochlorothiazide or indapamide or Natrilix or furosemide or frusemide or Lasix or Lipitor or Simvador or bezafibrate or Bezalip or ezetimibe or Ezetrol or fenofibrate or Supralip or Clonidine or orlistat or loperamide or adalat or amlodipine or bendrofluazide or bisoprolol or candesartan cilexetil or diltiazem or enalapril or metoprolol or perindopril or diclofenac or ibuprofen or meloxicam or naproxen or cerazette or elleste duet or kliovance or premarin or fybogel orange or lactulose product or movicol oral powder or senna or aspirin or ????????statin or ??????????prazole or metformin).mp. [mp=title, abstract, original title, name of substance word, subject heading word, floating sub-heading word, keyword heading word, organism supplementary concept word, protocol supplementary concept word, rare disease supplementary concept word, unique identifier, synonyms] | |
| 8 | 6 or 7 | |
| 9 | exp Risk Factors/ or exp INCIDENCE/ | |
| 10 | (Association* or effect or effects or incidence or risk* or incident* or rate ratio* or hazard* or odds ratio*).mp. [mp=title, abstract, original title, name of substance word, subject heading word, floating sub-heading word, keyword heading word, organism supplementary concept word, protocol supplementary concept word, rare disease supplementary concept word, unique identifier, synonyms] | |
| 11 | 9 or 10 | |
| 12 | 5 and 8 and 11 | |
| 13 | Randomized Controlled Trial/ or Randomized Controlled Trials as Topic/ or Random Allocation/ or Double Blind Method/ or Single Blind Method/ or clinical trial/ or exp Clinical Trials as topic/ | |
| 14 | clinical trial, phase i.pt. or clinical trial, phase ii.pt. or clinical trial, phase iii.pt. or clinical trial, phase iv.pt. or controlled clinical trial.pt. or randomized controlled trial.pt. or multicenter study.pt. or clinical trial.pt. | |
| 15 | 13 or 14 | |
| 16 | PLACEBOS/ | |
| 17 | (clinical adj trial$).tw. or ((singl$ or doubl$ or treb$ or tripl$) adj (blind$3 or mask$3)).tw. or placebo$.tw. or randomly allocated.tw. or (allocated adj2 random$).tw. | |
| 18 | 16 or 17 | |
| 19 | 15 or 18 | |
| 20 | case report.tw. | |
| 21 | letter/ or historical article/ | |
| 22 | 20 or 21 | |
| 23 | 19 not 22 | |
| 24 | exp Cohort Studies/ or exp case-control studies/ | |
| 25 | epidemiologic methods/ | |
| 26 | limit 26 to yr=1966-1989 | |
| 27 | cohort$.tw. or controlled clinical trial.pt. or (case$ and control$).tw. | |
| 28 | 24 or 26 or 27 | |
| 29 | exp Cross-Sectional Studies/ | |
| 30 | 24 not 29 | |
| 31 | exp animals/ not humans.sh. | |
| 32 | 23 or 30 | |
| 33 | 12 and 32 | |
| 34 | 33 not 31 | |

**Appendix table 2** Summary of included studies investigating non-steroidal anti-inflammatory drugs (NSAIDs) by study design

| **Paper** | **Country [Data source]** | **Inclusion/Exclusion criteria** | **Timing of exposure** | **Exposure definition** | **Exposure ascertainment** | **Outcome (proximal colon) definition** | **Outcome ascertainment** | **Number included in analysis** | **Follow-up in years: Mean (SD) unless otherwise specified^a^** | **Analyses (covariates included in models in publication)** | **Number of proximal colon cancer cases** | **Results**  **RR/HR/OR (95% CI)** |
| --- | --- | --- | --- | --- | --- | --- | --- | --- | --- | --- | --- | --- |
| ***Randomised Controlled Trials*** | | | | | | | | | | | | |
| Cook 2013 [70] | United States  [Women’s Health Study (WHS)] | - Inclusion: Female health professionals ≥45 years, not taking aspirin or NSAIDs (or willing to cease use during study), anticoagulants, corticosteroids, supplements (vitamin A, E or beta carotene) more than once week - Exclusion: history of cancer (except for non-melanoma skin cancer), cardiovascular disease, or other major chronic illness, currently enrolled in the Nurses' Health Study and those who showed non-compliance during the study run-in period | 1993-2004 | Aspirin use (100 mg aspirin every other day) | Not Applicable | Not Reported | Self-reported questionnaire verified by review of medical records by a committee of physicians blinded to study outcome, death certificates, national death index | 39,866  (Aspirin: 19,934  Placebo:19,942) | 17.5, 10.4-18.8 (median, range) | Cox proportional hazards regression models; Intention to treat  (age, vitamin E use) | 208 | HR 0.73 (0.55-0.95) |
| Rothwell, 2010 [63] | United Kingdom [Thrombosis Prevention Trial (TPT);  British Doctors Aspirin Trial (BDAT);  United Kingdom Transient Ischaemic Attack (UK-TIA) Aspirin Trial | - Inclusion: TPT – men 45 to 69 years old with an increased risk of vascular events   UK-TIA - men and women over 40 years old with a recent transient ischaemic attack or minor ischaemic stroke  BDAT – male British doctors resident in the UK born on or after 1900 with no regular use of aspirin   - Exclusion: history of cancer at baseline - TPT – history of possible peptic ulceration, myocardial infarction, stroke, taking drugs incompatible with trial intervention - UK-TIA – history of disabling major stroke or underlying non-arterial thromboembolism causes, cerebrovascular event (3 months prior), severe intercurrent non-vascular disease. Those like to have side effect from aspirin or taking aspirin 90 days before randomisation, needing regular aspirin or antihaemostatic medication or for whom follow-up or compliance would be an issue - BDAT – history of aspirin intolerance, alcoholism, chronic renal failure, or peptic ulceration | TPT: 1989-1997  UK TIA: 1979-1986  BDAT: 1978-1984 | Aspirin use: 75 mg-1200 mg daily (TPT – 75 mg;  UK-TIA – 1200 mg or 300 mg;  BDAT – 500 mg)  Aspirin treatment duration: ≥ 5 years | Not Applicable | caecum to splenic flexure | cancer registry, death certificates | 301,240 | 18.3 (median) | Cox proportional hazards regression models; Intention to treat  (study) | 69 | All patients:  HR 0.45 (0.28-0.74)  75 mg versus placebo (TPT only): HR 0·49, (0·23–1·06)  Scheduled treatment duration ≥5 years: HR 0.35 (0.20-0.63), p<0.0001 |
| ***Observational – cohort studies*** | | | | | | | | | | | | |
| Allison, 2006 [60] | United States [Women's Health Initiative (WHI)] | - Inclusion: post-menopausal women between 50 to 79 years, able to give consent and likely to survive for 3 years - Exclusion: history of CRC or conditions reducing survival or medical complications (e.g. drug and alcohol addiction, dementia) | 1993-1998 | Aspirin use (yes)  Duration (0.1–0.9, 1–1.9, 2–2.9, 3–3.9, 4–4.9, and ≥5 years; 1–5, 5.1–10, 10.1–20, >20 years)  Dose (1–164, 165–300, 301–494, ≥495 mg; 1–324, ≥325 mg) | Interview-administrated questionnaire | caecum, ascending colon, hepatic flexure | Medical records and pathology reports | 91,574  (Aspirin: 20,083;  No aspirin: 71,491) | 6.4 (SD not reported) | Cox proportional hazards regression models  (age, BMI, waist/hip ratio, family history of CRC, history of colonoscopy or sigmoidoscopy, smoking status, ethnicity, postmenopausal hormone therapy, household income, physical activity, b-carotene, vitamin C, vitamin E, selenium, folic acid, calcium, fibre, saturated fat intake  NB: adjustments above reported for CRC, sub-site-specific covariates not explicitly specified) | 266 | No significant associations (no estimates presented) |
| Chan, 2008 [67] | United States [Health Professionals Follow-up Study (HPFS)] | - Inclusion: Male health professionals - Exclusion: a history of cancer (except non-melanoma skin cancer), inflammatory bowel disease, a familial polyposis syndrome, or implausible reported dietary data. | 1986 - 2004 | Aspirin use (non-regular use, regular use [consumption of 2 or more standard tablets per week]) | Self-administered questionnaire | proximal to splenic flexure | Self- or next-of-kin report verified with medical records and pathology reports, National Death Index. Cancer subsite determined by a review of medical records by a study physician blinded to exposure. | 47,363  (Regular users: 13,922;  Non-regular users: 33,441) | 16.1 (calculated) | Cox proportional hazards regression models  (age, smoking history (before age 30), BMI, regular vigorous exercise, CRC in a parent or sibling, history of endoscopy, history of polyp, current multivitamin use, beef, pork, or lamb as a main dish, alcohol consumption, and energy-adjusted quintiles of folate and calcium intake) | 315  (Regular users: 139; Non-regular users: 176) | RR: 0.80 (0.63–1.00) |
| Cheung, 2020 [68] | Hong Kong [Clinical Data Analysis and Reporting System (CDARS)] | - Inclusion: Men and women ≥40 with a colonoscopy between 2005 to 2014 in public hospitals - Exclusion: a history of CRC, inflammatory bowel disease, colectomy or CRC detected within 6 months of index colonoscopy | 2000-2013 | NSAIDs use (non-use, use [≥90 days prior to index colonoscopy]) | Electronic healthcare database | Proximal colon cancer within 6 months to 3 years of colonoscopy  Anatomic definition: caecum to transverse colon [ICD-9 codes 153.4, 153.6, 153.0, 153.1] | Electronic healthcare database | 187,897 (NSAID users: 21,757;  NSAID nonusers: 166,140)  Proximal colon cancer analysis specifically: 187,190 | 2.98 (calculated) | Cox proportional hazards model; propensity score regression adjustment  (age at index colonoscopy, sex, history of colonic polyps, polypectomy at index colonoscopy, smoking status, alcohol consumption, diabetes mellitus, hypertension, dyslipidaemia, atrial fibrillation, ischaemic heart disease, congestive heart failure, stroke, chronic renal failure, cirrhosis, dementia, parkinsonism, concurrent medications (aspirin, cyclooxygenase-2 inhibitors, statins), annual centre endoscopy volume and centre polypectomy rate) | 147 | HR: 0.48 (0.24-0.95)  Duration:  ≤1 year: HR 0.53 (0.45-0.63)  >1 year: HR 0.43 (0.26-0.70) |
| Larrson, 2006 [66] | Sweden - Vastmanland, Orebro, Uppsala  [Swedish Mammography Cohort, Cohort of Swedish Men] | - Inclusion: Women – all female residents in Vastmanland and Uppsala counties born between 1914 and 1948. Men - all male residents of Vastmanland and Orebro counties born between 1918 and 1952 - Exclusion: a history of cancer (except non-melanoma skin cancer), no baseline questionnaire, missing aspirin use data, missing or incorrect national registration number. | 1997 | Aspirin use (more than six tablets per week) | Self-administered questionnaire | caecum, ascending colon, or transverse colon | Computer linkage to national and regional cancer registries and national death and population registers | 74,250  (Aspirin use: 32,135; No aspirin use: 42,115) | 7.2 (SD not reported) | Cox proportional hazards regression models  (age, education, family history of CRC, BMI, leisure-time physical activity, history of diabetes, smoking) | Not reported | RR: 0.82 (0.52–1.30) |
| Mahipal, 2006 [62] | United States – Iowa [Iowa Women’s Health Study] | - Inclusion: Women between the ages of 55 to 69 years resident in Iowa - Exclusion: history of cancer (except non-melanoma skin cancer) or missing data (e.g. aspirin or NSAID use), or with a tumour across two subsites or without a definitive subsite | 1992 | Aspirin or non-aspirin NSAID use (≤1 per week, ≥2 per week) | Self-administered questionnaire | ICD-O-2 codes: C18.0-C18.5 [caecum to splenic flexure] | Cancer registry, death index | 27,160 | 9.7 (calculated) | Cox proportional hazards regression models  (age, BMI, waist-to-hip ratio, calcium intake, multivitamin use, estrogen use, family history of colon cancer, physical activity, smoking status, aspirin use [for non-aspirin NSAID use analyses], non-aspirin NSAID use [for aspirin use analyses]) | Aspirin use: 365  Non-aspirin NSAID use: 365 | Aspirin:  ≤1 per week: HR 0.78 (0.60-1.02)  ≥2 per week: HR 0.67 (0.51-0.87)  Non-aspirin NSAID:  ≤1 per week: HR 1.10 (0.83-1.46)  ≥2 per week: HR 0.71 (0.52-0.97)  Effect of aspirin by category of non-aspirin NSAID use:  Never non-aspirin NSAID:  ≤1 per week: HR 0.81 (0.58-1.12)  ≥2 per week: HR 0.65 (0.46-0.90)  ≤1 weekly non-aspirin NSAID:  Never: HR 1.26 (0.76-2.09)  ≤1 per week: HR 0.90 (0.59-1.37)  ≥2 per week: HR 0.59 (0.34-1.01)  ≥2 weekly non-aspirin NSAID:  Never: HR 0.62 (0.37-1.03)  ≤1 per week: HR 0.40 (0.20-0.81)  ≥2 per week: HR 0.65 (0.40-1.03)  *Non-stratified crude (univariate) estimates (calculated by authors from the number of events and person-time reported in article).*  Aspirin:  Current vs never use: RR: 0.78 (0.63-0.98)  Non-aspirin NSAID:  Current vs never use: RR: 0.83 (0.67-1.03) |
| Murphy, 2018 [51] | Europe – [Denmark, France, Germany, Greece, Italy, Netherlands, Norway, Spain, Sweden, UK [European Prospective Investigation into Cancer and Nutrition (EPIC)] | - Inclusion: Healthy men and women between the ages of 35-74 years. - Exclusion: History of cancer, missing data (relevant medication use, alcohol consumption or follow-up) or those in extreme 1% of energy intake to energy requirement ratio | 1992-2000 | NSAID use (yes) | Self-administered questionnaire | ICD-O-2 codes: C18.0-C18.5 [caecum to splenic flexure] | Cancer registries or combination of insurance records, cancer and pathology registries and active follow-up | 476,160 | 14.9 (median) | Cox proportional hazards regression models; competing risk analysis – joint method; heterogeneity by subsite using likelihood ratio test  (sex, centre, age at recruitment adjusted for BMI, height, physical activity, smoking status and intensity, education level, ever use of menopausal hormone therapy, intake of alcohol, red and processed meats, calcium, fibre) | 693 | HR: 0.81 (0.61-1.06) |
| Tabung, 2017 [64] | United States [Women’s Health Initiative (WHI)] | - Inclusion: post-menopausal women between 50-79 years, able to give consent and likely to survive for 3 years - Exclusion: history of prevalent cancer, missing data on CRC status, any cancer diagnosis or second primary CRC diagnosis within 3 years of follow-up. Those with extreme energy intake or BMI values or only one questionnaire completed. | 1993-1998 | NSAID use (regular - at least two times per week in the past two weeks) | Self-administered questionnaire | ICD-O-2 codes: C18.0, C18.2–18.4 [caecum to transverse colon – excluding appendix] | Self-report, verification with medical records and pathology reports | 87,042 | 16.2 (median) | Cox proportional hazards regression models  (age, race/ethnicity, educational level, smoking status, diabetes, hypertension, arthritis, category and duration of estrogen use, category and duration of estrogen and progesterone use, BMI, physical activity, total energy intake) | 552 | Regular Users of NSAIDs  Patterns of Change in Dietary Inflammatory Potential  Anti-inflammatory change: HR 1.13 (0.78, 1.64)  Neutral inflammation stable: HR 1.28 (0.92-1.77)  Proinflammatory change: HR 1.40 (0.98-2.00)  Proinflammatory stable: HR 0.74 (0.53-1.02)  Reference Q1 (More anti-inflammatory diet)  Q2: HR 1.35 (0.96-1.90)  Q3: HR 1.24 (0.87- 1.76)  Q4: HR 0.87 (0.59- 1.29)  Q5 (more proinflammatory diet): HR 0.91 (0.60-1.37)  p-trend 0.10  *Non-stratified crude (univariate) estimates (calculated by authors from the number of events and number of participants reported in article).*  NSAID  regular use vs no use: RR: 0.99 (0.84-1.17) |
| Wang, 2015 [65] | United States – Seattle-Puget Sound [VITamins And Lifestyle (VITAL) study] | - Inclusion: Men and women between the ages of 50-76 and resident in are covered by Seattle-Puget Sound cancer registry - Exclusion: history of or missing data on history of CRC, ulcerative colitis, Crohn's disease or intestinal polyposis, missing data on NSAID use | 1990-2002 | NSAIDs use (low use [<4 days/week or <4 years], high use [>4 days/week and >4 years]) | Self-administered questionnaire | ICD-O-3 codes: C18.0–C18.5 [caecum to splenic flexure] | Cancer and death registries, post office database | 73,458 | 8.4 (SD not Reported) | Cox proportional hazards regression models  (age, gender, race, education, BMI, MET hours per week of moderate/vigorous activity, smoking, alcohol intake, fruit and vegetable intake, red meat intake, dietary and supplemental calcium intake, fibre intake, first-degree family history of CRC, screening history, female hormone replacement therapy use, coronary artery disease, frequent headache, arthritis or joint pain, diabetes, cholesterol-lowing drug use) | 139 | NSAID low use: HR 0.65 (0.44-0.97)  NSAID high use: HR 0.44 (0.27-0.70)  p-trend 0.062  *Non-stratified crude (univariate) estimates (calculated by authors from the number of events and number of participants reported in article).*  NSAID use within last 10 years vs no use: RR: 0.65 (0.47-0.90) |
| Wei, 2017 [57] | United States [Nurses' Health Study (NHS)] | - Inclusion: Married female registered nurses born between the ages of 30 to 55 and living in one of 11 US states - Exclusion: a history of cancer. missing date of birth, height or weight at 18 years, dietary and lifestyle values outside of a feasible range (generalised “extreme Studentized deviate” many-outlier detection method) | 1980-2010 | Aspirin use (never, 7 tablets per week per year) | Self-administered questionnaire | caecum, ascending colon, or transverse colon | Self-administered questionnaire. Diagnosis confirmed by medical record review. | 90,286 | Not reported | Cox proportional hazards regression models (competing risks model)  (age, dietary intake of folate, calcium, alcohol, red and processed meats, family history of CRC in a first-degree relative, cumulative smoking history, BMI, leisure-time physical activity, height, history of colorectal screening by endoscopy, aspirin use) | 821 | HR: 0.75 (0.64-0.87) |
| ***Observational – nested case-control studies*** | | | | | | | | | | | | |
| Demb, 2019 [69] | United States [US Veterans Health Administration (VHA)] | - Inclusion: US veterans with ≥1 colonoscopy during 1999 to 2011; Cases - and with CRC diagnosed 6 months before or after of baseline colonoscopy; Controls – and with no prior CRC and a normal baseline colonoscopy - Exclusion: Cases - unknown SEER stage, carcinoma in situ, or non-adenocarcinoma histology; Controls – CRC diagnosis within 3 years of baseline colonoscopy | 1998-2010 | Aspirin use: ≥2 prescriptions or ≥2 mentions of aspirin up to 1 year prior to colonoscopy | Electronic health records | ICD-O-3 codes: C18.0, C18.2–C18.4 [caecum, ascending colon, hepatic flexure or transverse colon] | Cancer registry, vital statistics registry | 21,744 CRC cases; 612,646 controls | Not applicable | Multinomial logistic regression (age, race/ethnicity, BMI, height, diabetes, smoking status) | 7,688  Among aspirin users: 3,008 | OR: 0.91 (0.86-0.95) |
| Friis, 2015 [61] | Denmark - North Jutland, Aarhus, Viborg, Ringkoebing [Danish registries] | - Inclusion: Men and women between 30-85 years of age, resident in Denmark - Exclusion: history of cancer (except non-melanoma skin cancer), inflammatory bowel disease, or familial adenomatous polyposis | from 1989,1996 or 1998 to 2011 (based on region) | Low-dose aspirin duration (<5 years, ≥5 years, ≥5 years high intensity - 150-mg tablets during exposure period)  Non-aspirin NSAIDs duration (<5 years, ≥5 years, ≥5 years high intensity - average dose per day, ≥0.3 defined daily dose during exposure period) | Prescription Database | ICD-10 codes: C18.0-C18.4 [caecum to transverse colon] | Cancer and vital statistics registries | Cases: 10,280  Controls: 102,800 | Not applicable | Conditional logistic regression models  (Low-dose aspirin/non-aspirin NSAID analyses: age, sex, area [by design], use of high-dose aspirin, HRT, antidepressants, and statins, history of diabetes mellitus, alcoholism, migraine, chronic obstructive pulmonary disease or asthma, history of cholecystectomy and colonoscopy  Low-dose aspirin analyses: non-aspirin NSAID use, rheumatic, soft tissue, or connective tissue disease  Non-aspirin NSAID analyses: low-dose aspirin use) | Aspirin: 2,700  Non-aspirin NSAID: 2,777 | Aspirin  <5 years: OR 1.03 (0.91–1.17)  ≥5 years: OR 0.94 (0.80–1.11)  ≥5 years, high-intensity: OR 1.06 (0.80–1.41)  Non-aspirin NSAID  <5 years: OR 1.00 (0.90–1.10)  ≥5 years: OR 0.91 (0.82–1.01)  ≥5 years, high-intensity: OR 0.80 (0.65-0.98)  *Non-stratified crude (univariate) estimates (calculated by authors from the number of events and number of participants reported in article).*  Aspirin:  Aspirin use vs non use: OR: 0.97 (0.88-1.07)  Non-aspirin NSAID  Non-aspirin NSAID use vs non use: OR: 0.95 (0.88-1.03) |

^a^ Follow-up calculated if not given in years (calculated by dividing person-years by number included in analysis)

BDAT= British Doctors Aspirin Trial; BMI=Body Mass Index; CDARS= Clinical Data Analysis and Reporting System; CI=Confidence Interval; CRC=colorectal cancer; EPIC=European Prospective Investigation into Cancer and Nutrition; HPFS= Health Professionals Follow-up Study; HR=Hazard ratio; HRT=Hormone Replacement Therapy; ICD-9=International Classification of Diseases volume 9; ICD-10=International Classification of Diseases volume 10; ICD-O-2=International Classification of Diseases for Oncology volume 2; ICD-O-3=International Classification of Diseases for Oncology volume 3; MET=Metabolic Equivalent of Task; NHS=Nurses’ Health Study; NSAID=Non-steroidal anti-inflammatory drug; OR=Odds Ratio; RR=Risk/Rate Ratio; SD=standard deviation; TPT= Thrombosis Prevention Trial; UK TIA=United Kingdom Transient Ischaemic Attack; VITAL=Vitamins And Lifestyle study; VHA=Veterans Health Administration; WHI=Women's Health Initiative; WHS=Women’s Health Study

**Appendix table 3** Summary of included cohort studies investigating exogenous hormones (hormone replacement therapy [HRT] and oral contraceptive [OC]) by medication type

| **Paper** | **Country [Data source]** | **Inclusion/Exclusion criteria** | **Timing of exposure** | **Exposure definition** | **Exposure ascertainment** | **Outcome (proximal colon) definition** | **Outcome ascertainment** | **Number included in analysis** | **Follow-up in years: Mean (SD) unless otherwise specified^a^** | **Analyses (covariates included in models)** | **Number of proximal colon cancer cases** | | **Results**  **RR/HR (95% CI)** |
| --- | --- | --- | --- | --- | --- | --- | --- | --- | --- | --- | --- | --- | --- |
| ***Postmenopausal Replacement Therapy or Hormone Replacement Therapy*** | | | | | | | | | | | | | |
| Buron Pust, 2017 [48] | United Kingdom [Million Women Study] | - Inclusion: Women between the ages of 50-64 invited for breast cancer screening in the UK - Exclusion: history of invasive cancer (except non-melanoma skin cancer) | 1996-2001 | HRT use (never, ever) | Self-administered questionnaire | ICD-10 codes: C18.0-C18.4 [caecum to transverse colon] | Cancer and vital statistics registries | 1,310,390 | 13.8 (3.4) | Cox proportional hazards regression models; test for heterogeneity; correction for multiple comparisons - Holm-Bonferroni method  (geographical region, socioeconomic status, height, BMI, smoking, alcohol, strenuous exercise, age at menarche, births, hysterectomy, sterilisation, age at menopause, OC use) | 6,278 | | RR 0.96 (0.89-1.03) |
| Henderson, 2010 [56] | United States – California [California Teachers Study] | - Inclusion: Past and current female public-school teachers and administrators and member of the California State Teachers Retirement System in 1995 - Exclusion: non-California resident, >80 years, limited participation in breast cancer research, history or unknown history of CRC, premenopausal or unknown menopausal status, hormone therapy or progestin-only users | 1995-2000 | HRT use (current, never) | Self-administered questionnaire | ICD-O-2 codes: C18.0–C18.5 [caecum to splenic flexure] | Linkage to state cancer registry | 56,864  (2,245 perimenopausal; 54,619 postmenopausal) | Not reported | Cox proportional hazards models  (race/ethnicity, BMI, physical activity, age at baseline) | 321 | | RR 0.67 (0.52- 0.87) |
| Hildebrand, 2009 [49] | United States [Cancer Prevention Study II (CPS-II) Nutrition Cohort] | - Inclusion: Postmenopausal women between the ages of 50-74 years - Exclusion: history of invasive cancer (except non-melanoma skin cancer) | 1992-2005 | HRT use (never, former, current)  HRT type (Estrogen-only, Estrogen plus Progesterone) | Self-administered questionnaire | ICD-O codes: C18.0, C18.2-C18.5 [caecum to splenic flexure – excluding appendix] | Self-reported questionnaire, verification with medical records or cancer registry, death certificates | 67,412 | 13.2 (not reported if mean, median or maximum) | Cox proportional hazards regression models  (age at first survey, time-dependent CRC endoscopy screening, BMI, smoking, education, race, physical activity, use of NSAIDs, use of multivitamins, red meat intake, family history of CRC, hysterectomy, type of menopause) | Estrogen-only: 248  Estrogen plus Progesterone: 224 | | Use (Former)  Estrogen-only: RR 1.05 (0.79-1.39)  Estrogen plus Progesterone: RR 1.05 (0.61-1.80)  Use (Current)  Estrogen-only: RR 0.76 (0.53-1.10)  Estrogen plus Progesterone: RR 1.16 (0.78-1.74) |
| Morois, 2012 [50] | France, [E3N cohort study (Etude Epidémiologique auprès de femmes de la Mutuelle Générale de l'Education Nationale)] | - Inclusion: Post-menopausal women between the ages of 40-6, living in France and with an insurance plan from Mutuelle Générale de l'Éducation Nationale (MGEN) - Exclusion: history of cancer other than basal cell carcinoma or in situ colorectal adenocarcinoma, no follow-up or lifetime HRT use data | 1992-2008 | HRT use (never, ever)  HRT type (never, Estrogen-only, Estrogen and Progestagen, Weak Estrogen, other HRT) | Self-administered questionnaire | caecum, ascending colon, hepatic flexure, transverse colon | Self-reported questionnaire verified with pathology reports, deaths from family report, insurance company, patient physician | 77,375 | 11.3 (4.6) (median, SD) | Cox proportional hazards regression models; test for heterogeneity Wald Chi-squared tests  (age, BMI, total physical activity, smoking status, CRC in first degree relatives, educational level) | Any HRT: 142  Estrogen-only: 80  Estrogen plus Progesterone: 126 | | Use  Ever: HR 0.83 (0.59–1.17)  Type used  Estrogen-only: HR 1.02 (0.66–1.58)  Estrogen and Progestagen: HR 0.90 (0.63–1.27)  Weak Estrogen: HR 0.96 (0.56–1.66)  Other HRT: HR 0.85 (0.49–1.49) |
| Murphy, 2018 [51] | Europe – [Denmark, France, Germany, Greece, Italy, Netherlands, Norway, Spain, Sweden, UK [European Prospective Investigation into Cancer and Nutrition (EPIC)] | - Inclusion: Healthy women between the ages of 35-74 years. - Exclusion: History of cancer, missing data (relevant medication use, alcohol consumption or follow-up) or those in extreme 1% of energy intake to energy requirement ratio | 1992-2000 | HRT use (never, ever, <2, 2 to <5, 5 to <8, 8 years) | Self-administered questionnaire | ICD-O-2 codes: C18.0-C18.5 [caecum to splenic flexure] | Cancer registries or combination of insurance records, cancer and pathology registries and active follow-up | 476,160 | 14.9 (median) | Cox proportional hazards regression models; competing risk analysis – joint method; heterogeneity by subsite using likelihood ratio test  (alcohol consumption, physical activity, and smoking status, age, sex, centre, age at recruitment, BMI, height, education level, intakes of alcohol, red and processed meats, calcium, fibre) | 1,100 | | Use  Ever: HR 0.95 (0.83,1.09)  Duration of use  <2 years: HR 0.91 (0.81,1.21)  2-<5 years: HR 0.97 (0.73,1.13)  5-<8 years: HR 1.11 (0.84,1.45)  ≥8 years: HR 0.81 (0.61,1.07) |
| Paganini-Hill, 1999 [52] | United States - Southern California [Leisure World Cohort] | - Inclusion: Residents of Southern California who owned homes in Leisure World Laguna Hills - Exclusion: history of cancer and/or no information on HRT use | 1981-1985 | HRT use (never, recent [use within one year of study entry]) | Self-administered questionnaire | Not Reported | Hospital pathology and tumour registries, self-report, hospital admissions, deaths from death certificates, national and commercial death indices | 7,701 | 11.1 (calculated) | Poisson regression analyses  (BMI, alcohol consumption, exercise, vitamin C intake, daily aspirin use, type of menopause, age at last menstrual period,  previous hemoccult, regular physician visits) | Not reported | | RR 0.75 (0.38-1.48) |
| Risch, 1995 [53] | Canada – Saskatchewan [Saskatchewan Health Plan Databases] | - Inclusion: Women between the ages of 43-49 years residing in Saskatchewan in 1976 and listed in the Saskatchewan Health master registration file. - Exclusion: History of diagnosis of CRC prior to 1976 | 1976-1987 | HRT use (never, ever)  HRT type (never, estrogens, progestins, combined estrogen-progestin use) | Prescription Drug Plan Database | Caecum to splenic flexure | cancer registry, active follow-up, death certificates, hospital reports, health plan registration databases | 32,973 | 13.7 (calculated) | Cox proportional hazards regression models  (age) | 55 | | Use  Any: RR 1.42 (0.79-2.55)  NB: Stratified analysis not included in forest plot since indistinguishable whether use was for OC or HRT. |
| Troisi, 1997 [54] | United States [Breast Cancer Detection Demonstration Project (BCDDP)] | - Inclusion: Postmenopausal women who during breast cancer screening, received a diagnosis of breast cancer, underwent surgery with no evidence of malignant breast cancer, were recommended for surgical consultation but without diagnostic intervention or had neither of the aforementioned - Exclusion: history of breast or CRC | 1979-1986 | HRT use (never, ever)  HRT Recency (never, recent, former)  Years since cessation (never, <5 years, ≥5 years) | First stage: Telephone interview  Second stage: self-administered questionnaire | Caecum to splenic flexure | Self-reported questionnaire, verification with pathology report, death certificate | 40,464 | 7.7 (SD not reported) | Poisson regression models  (age) | 70 | | Use  Recent: RR 1.5 (0.80-3.0)  Any estrogen**:** RR 1.7 (1.0-2.7)  Former: RR 1.7 (1.0-2.9)  Duration of use  Ever  <5 years: RR 1.8 (1.0-3.1)  ≥5 years: RR 1.5 (0.86-2.8)  Recent  <5 years: RR 2.3 (0.91-5.8)  ≥5 years: RR 1.2 (0.54-2.7)  Former  <5 years: RR 1.7 (0.93-3.1)  ≥5 years: RR 1.9 (0.95-3.7)  Years since cessation  <5 years RR 1.1 (0.47-2.8)  ≥5 years RR 2.0 (1.1-3.5)  Unopposed estrogen**:** RR 1.6 (1.0-2.7) |
| Wei, 2017 [57] | United States [Nurses' Health Study (NHS)] | - Inclusion: Postmenopausal married female registered nurses born between the ages of 30 to 55 and living in one of 11 US states - Exclusion: a history of cancer. missing date of birth, height or weight at 18 years, dietary and lifestyle values outside of a feasible range (generalised “extreme Studentized deviate” many-outlier detection method) | 1980-2010 | HRT use (never, past, current) | Self-administered questionnaire | caecum, ascending colon, or transverse colon | Self-administered questionnaire. Diagnosis confirmed by medical record review. | Number of postmenopausal women not reported | Not reported | Cox proportional hazards regression models (competing risks model)  (age, dietary intake of folate, calcium, alcohol, red and processed meats, family history of CRC in a first-degree relative, cumulative smoking history, BMI, leisure-time physical activity, height, history of colorectal screening by endoscopy, aspirin use) | 821 (number for all women, number in postmenopausal women only not reported) | | Current use:  HR: 0.95 (0.79-1.14)  Past use: HR: 0.98 (0.83-1.16) |
| ***Oral contraceptives*** | | | | | | | | | | | | | |
| Buron Pust, 2017 [48] | United Kingdom [Million Women Study] | - Inclusion: Women between the ages of 50-64 invited for breast cancer screening in the UK - Exclusion: history of invasive cancer (except non-melanoma skin cancer) | 1996-2001 | OC duration (never use, <5 years, ≥5 years) | Self-administered questionnaire | ICD-10 codes: C18.0-C18.4 [caecum to transverse colon] | Cancer and vital statistics registries | 1,310,390 | 13.8 (3.4) | Cox proportional hazards regression models; test for heterogeneity; correction for multiple comparisons - Holm-Bonferroni method  (geographical region, socioeconomic status, height, BMI, smoking, alcohol, strenuous exercise, age at menarche, births, hysterectomy, sterilisation, age at menopause, HRT use) | 6,278 | | Duration of use  <5 years: RR 1.05 (0.98-1.12)  ≥5 years: RR 1.08 (1.01-1.15)  Pooled estimate calculated and used in figures (random effects meta-analysis): 1.07 (1.02-1.12) |
| Charlton 2015 [58] | United States [Nurses' Health Study I (NHS I) & II (NHS II)] | - Inclusion: NHS I – female registered nurses between 30-55 years of age; NHS II – female registered nurses between 25-42 years of age - Exclusion: history of cancer, except for non-melanoma skin cancer, history of ulcerative colitis, non-completion of the baseline dietary questionnaire, diagnosis of cancer | NHSI 1980- 2010; NHSII 1991-2009 | OC use (never use, ever use [minimum 2 months]) | self-administered questionnaire | Not reported | CRC - self-reported questionnaire verified by review of medical and pathology reports. Deaths -reported by family members, postal system and National Death Index. CRC deaths verified by review of medical records. | NHS I: OC use 45,237; never use 43,454  NHS II: OC use 80,123; never use12,957 | NHS I: 30 years (max)  NHS II: 19 years (max) | Cox proportional hazards regression models; competing risk analysis  (age, BMI, height, physical activity, smoking, processed and red meat, folate, calcium, total energy, aspirin use, alcohol, age at first birth, parity, hormone therapy use, family history, and previous endoscopy screening) | NHS I: OC use 330; never use 493  NHS II: OC use 54; never use 14 | | NHS I: HR 1.14 (0.98-1.32); NHS II: HR 0.60 (0.35-1.15)  Duration of use:  NHS I:  ≤1 year: HR 1.34 (1.08–1.67)  >1 to <2 years: HR 1.20 (0.82–1.74)  ≥2 to <5 years: HR 0.89 (0.67–1.17)  ≥5 to <10 years: HR 1.19 (0.93–1.52)  10+ years: HR 1.17 (0.84–1.63)  p-trend 0.36  NHS II:  ≤1 year: HR 0.75 (0.33–1.69)  >1 to <5 years: HR 0.96 (0.53–1.74)  5+ years: HR 0.51 (0.26–1.00)  p-trend 0.05 |
| Lin, 2007 [55] | United States  [Women’s Health Study (WHS)] | - Inclusion: Female health professionals aged 45 years or older, with no intention to become pregnant and no previous history of cancer (except non-melanoma skin cancer) or heart disease. - Exclusion: unusable information on OC use or risk factors for CRC | 1992 | OC use (never, ever, duration [ <6, 6–<36, 36–<60, or ≥60 months]) | Self-administered questionnaire | Not reported | Self-report and verification by blinded physicians of outcomes from medical records and pathology reports | 39,680 | 11 (SD not reported) | Cox proportional hazards regression models  (age, randomized treatment assignment, family history of CRC in a first-degree relative, history of benign colorectal polyps, BMI, physical activity, smoking status, multivitamin use, alcohol consumption, baseline aspirin use, postmenopausal hormone therapy, and red meat consumption) | | Not reported | No significant associations found (data not shown) |
| Murphy, 2018 [51] | Europe – [Denmark, France, Germany, Greece, Italy, Netherlands, Norway, Spain, Sweden, UK [Prospective Investigation into Cancer and Nutrition (EPIC)] | - Inclusion: Healthy men and women between the ages of 35-74 years. - Exclusion: History of cancer, missing data (relevant medication use, alcohol consumption or follow-up) or those in extreme 1% of energy intake to energy requirement ratio | 1992-2000 | OC use (never, ever) | Self-administered questionnaire | ICD-O-2 codes: C18.0-C18.5 [caecum to splenic flexure] | Cancer registries or combination of insurance records, cancer and pathology registries and active follow-up | 476,160 | 14.9 (median) | Cox proportional hazards regression models; competing risk analysis – joint method; heterogeneity by subsite using likelihood ratio test  (alcohol consumption, physical activity, and smoking status, age, sex, centre, age at recruitment, BMI, height, education level, intakes of alcohol, red and processed meats, calcium, fibre, ever use of menopausal hormone therapy) | 1,178 | | HR 1.00 (0.88,1.14) |

^a^ Follow-up calculated if not given in years (calculated by dividing person-years by number included in analysis)

BCDDP= Breast Cancer Detection Demonstration Project; BMI=Body Mass Index; CI=Confidence Interval; CRC=colorectal cancer; CPS-II= Cancer Prevention Study II; EPIC=European Prospective Investigation into Cancer and Nutrition; E3N=Etude Epidémiologique auprès de femmes de la Mutuelle Générale de l'Education Nationale; HR=Hazard ratio; HRT=Hormone Replacement Therapy; ICD=International Classification of Diseases; ICD-7=International Classification of Diseases volume 7; ICD-10=International Classification of Diseases volume 10; ICD-O-2=International Classification of Diseases for Oncology volume 2; ICD-O-3=International Classification of Diseases for Oncology volume 3; MGEN=Mutuelle Générale de l'Éducation Nationale; NHS=Nurses’ Health Study; NSAID=Non-steroidal anti-inflammatory drug; OC=oral contraceptives; OR=Odds Ratio; RR= Risk/Rate Ratio; SD=Standard deviation; WHS=Women’s Health Study

**Appendix table 4** Summary of included cohort studies investigating statins

| **Paper** | **Country [Data source]** | **Inclusion/Exclusion criteria** | **Timing of exposure** | **Exposure definition** | **Exposure ascertainment** | **Outcome (proximal colon) definition** | **Outcome ascertainment** | **Number included in analysis** | **Follow-up in years: Mean (SD) unless otherwise specified^a^** | **Analyses (covariates included in models)** | **Number of proximal colon cancer cases** | **Results**  **RR/HR (95% CI)** |
| --- | --- | --- | --- | --- | --- | --- | --- | --- | --- | --- | --- | --- |
| Cheung 2019 [71] | Hong Kong [Clinical Data Analysis and Reporting System (CDARS)] | - Inclusion: Men and women ≥40 with a colonoscopy between 2005 to 2013 in public hospitals   Exclusion: a history of CRC, inflammatory bowel disease, colectomy or CRC detected within 6 months of index colonoscopy | 2000-2013 | Statin use (non-use, use [≥90 days prior to index colonoscopy]) | Electronic healthcare database | Proximal colon cancer within 6 months to 3 years of colonoscopy  Anatomic definition: caecum to transverse colon [ICD-9 codes 153.4, 153.6, 153.0, 153.1] | Electronic healthcare database | Proximal colon cancer analysis specifically: 47,775 (Statin users: 17,601;  Non-statin users: 30,174) | 2.98 (calculated) | Competing risk regression model; propensity score matching  (age at index colonoscopy, sex, history of colonic polyps, polypectomy at index colonoscopy, smoking, heavy alcohol consumption, diabetes mellitus, hypertension, dyslipidaemia, atrial fibrillation, ischaemic heart disease, congestive heart failure, stroke, chronic renal failure, cirrhosis, dementia, parkinsonism, concurrent use of aspirin, cyclooxygenase-2 inhibitors or non-steroidal anti-inflammatory drugs, annual centre endoscopy volume and centre polypectomy rate) | 62 (Statin users: 14;  Non-statin users: 48) | Subdistribution HR: 0.50 (0.28-0.91) |
| Jacobs, 2006 [72] | United States [Cancer Prevention Study II (CPS-II) Nutrition Cohort] | - Inclusion: Men and women between 50-74 years and resident in one of 21 states with population-based cancer registries with 90% ascertainment of cases. - Exclusion: history of CRC, lost to follow-up, incomplete information | 1997-1999 | Cholesterol-Lowering Drug use as proxy for statins (former use, current use <5 years, current use ≥5 years) | Self-administered questionnaire | Not reported | Self-reported questionnaire verified with medical records or cancer registry or death index | 132,136 | Not reported | Cox proportional hazards regression models  (age, sex, race, BMI, education, physical activity level, HRT, red meat consumption, NSAID use, endoscopy history, elevated cholesterol, history of heart attack) | Not reported | Use:  no association (no estimates presented) |
| Lee, 2011 [73] | United States  [Nurses' Health Study (NHS),  Health Professionals Follow-up Study (HPFS)] | - Inclusion: NHS – female registered nurses between 30-55 years; HPFS – male health professionals between 40-75 years - Exclusion: history of cancer (except non-melanoma skin cancer), history of ulcerative colitis. missing exposure data | NHS: 1994-2002  HPFS: 1990 - 2002 | Statin use (non-use, current) | Self-administered questionnaire | Not Reported | Self-reported questionnaire verified with medical records, death index | NHS: 91,155  HPFS: 4  0,767 | 12.8 (calculated) | Cox proportional hazards regression models; competing risk analysis – duplication method; heterogeneity by subsite using likelihood ratio test  (age, calendar year, study, pack-years of smoking before 30, aspirin dose, height, BMI, family history of CRC, history of endoscopy, red meat intake, alcohol intake, total energy intake) | Not reported | Current use:  RR 1.14 (0.93–1.39) |
| Lee, 2019 [74] | Korea (National Health Insurance Service-Health Screening [NHIS-HEALS] cohort) | - Inclusion: Men and women between the ages of 40-79 years enrolled in a national health screening programme and with total cholesterol ≥250 mg/dL or prescribed anti-dyslipidaemia medications between 2002-2003 - Exclusion: cancer diagnosis between 2002-2004, ischaemic heart disease or cerebrovascular disease between 2002-2003, death between 2002-2004 or incomplete data on confounders | 2002-2003 | Statin use (non-use [between 2002-2015], low use, high use) | Not reported | ICD-10 codes C18.0-C18.5 [caecum to splenic flexure] | Not reported | 31,149 (Statin users: Low use – 8,868; High use - 8,869  Non-statin users: 13,412) | 12.7 (median) | Cox proportional hazards model  (age, sex, BMI, systolic blood pressure, glucose, total cholesterol, income status, smoking status, alcohol consumption, physical activity, diabetes, hypertension) | Not reported | Low-users:  HR 0.61 (0.36-1.04)  High-users:  HR 0.30 (0.16-0.57)  Pooled estimate calculated and used in figures (random effects meta-analysis): 0.43 (0.22-0.88) |
| Simon, 2012 [75] | United States [Women’s Health Initiative (WHI)] | - Inclusion: Post-menopausal women between the age of 50-79 able to provide informed consent and remain resident and alive for least 3 years - Exclusion: a history of or an unknown history of CRC or previous statin use unknown | 1993-1998 | Statin use (non-use, current use [at interview]) | Interview-administrated questionnaire, confirmed by visual inspection of medications | Not reported | Self-report and verification with medical records and pathology reports | 159,219 (Statin use: 12,030; Non-use: 147,189) | 10.7 (2.9) | Cox proportional hazards  (age, ethnicity, education, smoking, alcohol use, physical activity, BMI, percent energy from fat, fruit and vegetable intake, dietary calcium, calcium supplement use, selenium supplement use, current healthcare provider, last medical visit within one year, colon screening at baseline, current hormone therapy use, family history of CRC, history of colon polyp removal, use of NSAIDs, hypertension, history of stroke and history of coronary artery disease) | 1,051 (Statin use: 90; Non-use: 961) | HR: 1.04 (0.81-1.33) |

^a^ Follow-up calculated if not given in years (calculated by dividing person-years by number included in analysis)

BMI=Body Mass Index; CDARS= Clinical Data Analysis and Reporting System; CI=Confidence Interval; CPS-II=Cancer Prevention Study II; CRC=colorectal cancer; HPFS=Health Professionals Follow-up Study; HR=Hazard ratio; HRT=Hormone Replacement Therapy; ICD-9=International Classification of Diseases volume 9; ICD-10=International Classification of Diseases volume 10; NHIS-HEALS= National Health Insurance Service-Health Screening; NHS=Nurses’ Health Study; NSAID=Non-steroidal anti-inflammatory drug; RR= Risk/Rate Ratio; SEER=Surveillance, Epidemiology, and End Results; SD=standard deviation; WHI=Women’s Health Initiative

**Appendix table 5** Summary of included nested case control study investigating metformin

| **Paper** | **Country [Data source]** | **Inclusion/Exclusion criteria** | **Timing of exposure** | **Exposure definition** | **Exposure ascertainment** | **Outcome (proximal colon) definition** | **Outcome ascertainment** | **Number included in analysis** | **Follow-up in years: Mean (SD) unless otherwise specified** | **Analyses (covariates included in models)** | **Number of proximal colon cancer cases** | **Results**  **RR/HR/OR (95% CI)** |
| --- | --- | --- | --- | --- | --- | --- | --- | --- | --- | --- | --- | --- |
| Demb, 2019 [59] | United States [Department of Veterans Affairs] | - Inclusion: US veterans with ≥1 colonoscopy during 1999 to 2014 with Type II diabetes; Cases - and with CRC diagnosed 6 months before or after of baseline colonoscopy; Controls – and with no prior CRC and a normal baseline colonoscopy - Exclusion: Cases - patients with inflammatory bowel disease or a diagnosis at or within 6 months of baseline colonoscopy, a history of CRC, unknown SEER stage, carcinoma in situ, or non-adenocarcinoma histology; Controls – CRC diagnosis within 3 years of baseline colonoscopy | 1998-2013 | Metformin use (non-use, current use [2 prescriptions ≤1 year preceding baseline colonoscopy]) | Electronic health records | ICD-O-3 codes: C18.0, C18.2–C18.4 [caecum, ascending colon, hepatic flexure or transverse colon] | Cancer registry, vital statistics registry | 6,650 diabetic CRC cases; 454,507 diabetic controls | Not applicable | Multivariable multinomial logistic regression  (age, race/ethnicity, BMI, smoking status, aspirin exposure) | 2,625  Among metformin users: 1,017 | OR: 0.96 (0.88-1.04) |

BMI=Body Mass Index; CI=Confidence Interval; CRC=colorectal cancer; HRT=Hormone Replacement Therapy; ICD-O-3=International Classification of Diseases for Oncology volume 3; NSAID=Non-steroidal anti-inflammatory drug; OR=Odds ratio; SD=standard deviation

**Appendix table 6** Summary of included cohort study investigating anti-hypertensive drugs

| **Paper** | **Country [Data source]** | **Inclusion/Exclusion criteria** | **Timing of exposure** | **Exposure definition** | **Exposure ascertainment** | **Outcome (proximal colon) definition** | **Outcome ascertainment** | **Number included in analysis** | **Follow-up in years: Mean (SD) unless otherwise specified^a^** | **Analyses (covariates included in models)** | **Number of proximal colon cancer cases** | **Results**  **HR (95% CI)** |
| --- | --- | --- | --- | --- | --- | --- | --- | --- | --- | --- | --- | --- |
| Cheung 2020 [47] | Hong Kong [Clinical Data Analysis and Reporting System (CDARS)] | - Inclusion: Men and women ≥40 with a colonoscopy between 2005 to 2013 in public hospitals   Exclusion: a history of CRC, inflammatory bowel disease, colectomy or CRC detected within 6 months of index colonoscopy | 2000-2013 | ACE inhibitor/ARB use (non-use, use [≥180 days up to 5 years prior to index colonoscopy] | Electronic healthcare database | Proximal colon cancer within 6 months to 3 years of colonoscopy  Anatomic definition:  caecum to transverse colon [ICD-9 codes 153.4, 153.6, 153.0, 153.1] | Electronic healthcare database | Proximal colon cancer analysis specifically: 187,190 (ACE/ARB users: 30,720;  Non- ACE/ARB users: 156,470) | 3.0 (calculated) | Cox proportional hazards model with PS regression adjustment  (age at index colonoscopy, sex, history of colonic polyps, polypectomy at index colonoscopy, smoking, alcohol consumption, diabetes mellitus, hypertension, dyslipidaemia, atrial fibrillation, ischaemic heart disease, congestive heart failure, stroke, chronic renal failure, cirrhosis, dementia, parkinsonism, concurrent use of aspirin, non-steroidal anti-inflammatory drugs or cyclooxygenase-2 inhibitors, annual centre endoscopy volume and centre polypectomy rate) | 147  (ACE/ARB users: 33;  Non- ACE/ARB users: 114) | Adjusted HR: 0.83 (0.51-1.35) |

^a^ Follow-up calculated if not given in years (calculated by dividing person-years by number included in analysis)

ACE=Angiotensin-converting enzyme; ARB=Angiotensin II Receptor Blockers; CDARS= Clinical Data Analysis and Reporting System; CI=Confidence Interval; CRC=colorectal cancer; HR=Hazard ratio; ICD-9= International Classification of Diseases volume 9; SD=standard deviation

**Appendix table 7: Risk of bias assessment of included publications**

| **Cohort studies (Newcastle Ottawa Scale)** | | | | | | | | | |
| --- | --- | --- | --- | --- | --- | --- | --- | --- | --- |
|  | **Representativeness of exposed cohort** | **Selection of non-exposed cohort** | **Exposure ascertainment** | **Outcome of interest not present at start of study** | **Comparability of cohorts^*^** | **Outcome assessment** | **Follow-up length (≥10 years)^**^** | **Adequacy of follow-up (≥90%)** | **Overall (score)** |
| Allison 2006 [60] | - | + | + | + | ++ | + | - | ? | **6** |
| Buron Pust 2017 [48] | - | + | - | + | ++ | + | + | + | **7** |
| Chan 2008 [67] | - | + | - | + | ++ | + | + | + | **7** |
| Charlton 2015 [58] | - | + | - | + | ++ | + | + | ? | **6** |
| Cheung 2019 ^***^ [71] | + | + | + | + | ++ | + | n/a | ? | **7/8** |
| Cheung 2020 (NSAIDs) ^***^ [68] | + | + | + | + | ++ | + | n/a | ? | **7/8** |
| Cheung 2020 ^***^ [47] | + | + | + | + | ++ | + | n/a | ? | **7/8** |
| Henderson 2010 [56] | - | + | - | + | ++ | + | + | + | **7** |
| Hildebrand 2009 [49] | - | + | - | + | ++ | + | + | + | **7** |
| Jacobs 2006 [72] | - | + | - | + | ++ | + | - | ? | **5** |
| Larsson 2006 [66] | + | + | - | + | ++ | + | - | + | **7** |
| Lee 2011 [73] | - | + | - | + | ++ | + | + | ? | **6** |
| Lee 2019 [74] | + | + | - | + | ++ | + | + | ? | **7** |
| Lin 2007 [55] | - | + | - | + | ++ | + | + | ? | **6** |
| Mahipal 2006 [62] | + | + | - | + | ++ | + | + | + | **8** |
| Morois 2012 [50] | - | + | - | + | ++ | + | + | + | **7** |
| Murphy 2018 [51] | - | + | - | + | ++ | + | + | + | **7** |
| Paganini-Hill 1999 [52] | - | + | - | + | ++ | + | + | + | **7** |
| Risch 1995 [53] | + | + | + | + | - | + | + | ? | **6** |
| Simon 2012 [75] | - | + | + | + | ++ | + | + | + | **8** |
| Tabung 2017 [64] | - | + | - | + | ++ | + | + | ? | **6** |
| Troisi 1997 [54] | - | + | - | + | - | + | - | - | **3** |
| Wang 2015 [65] | - | + | - | + | ++ | + | - | + | **6** |
| Wei 2017 [57] | - | + | - | + | ++ | + | + | ? | **6** |
| **Nested case-control studies (Newcastle Ottawa Scale)** | | | | | | | | | |
|  | **Adequacy of case definition** | **Representativeness of cases** | **Selection of controls** | **Definition of controls** | **Comparability of cases and controls *** | **Exposure ascertainment** | **Same method of ascertainment for cases and controls** | **Non-response rate (≤10%)** | **Overall (score)** |
| Demb 2019 (aspirin) [69] | + | + | + | + | ++ | + | + | + | **9** |
| Demb 2019 (metformin) [59] | + | + | + | + | ++ | + | + | + | **9** |
| Friis 2015 [61] | + | + | + | + | ++ | + | + | + | **9** |
| **Randomised controlled trials (Cochrane Collaboration tool)** | | | | | | | | | |
|  | **Randomisation process** | **Deviations from intended interventions** | **Missing outcome data** | **Outcome measurement** | **Selection of reported results** |  |  |  | **Overall** |
| Cook 2013 [70] | ? | + | + | + | + |  |  |  | **?** |
| Rothwell 2010 [63] | ? | + | + | + | + |  |  |  | **?** |

+ low risk of bias; - high risk of bias; ? unclear risk of bias (Newcastle Ottawa Scale)/some concerns (Cochrane Collaboration tool);

^*^ + controlled for age, sex (where relevant) and important confounders (e.g. BMI); ++ additionally controlled for relevant confounders

^**^ at least 10 years of follow-up possible from start of study

^***^ outcome was proximal colon cancer incidence within 3 years after index colonoscopy therefore follow-up length was not considered for risk of bias.

**Appendix figure 1** Percentage of studies meeting each criterion for the risk of bias assessment for cohort studies using the Newcastle Ottawa Scale


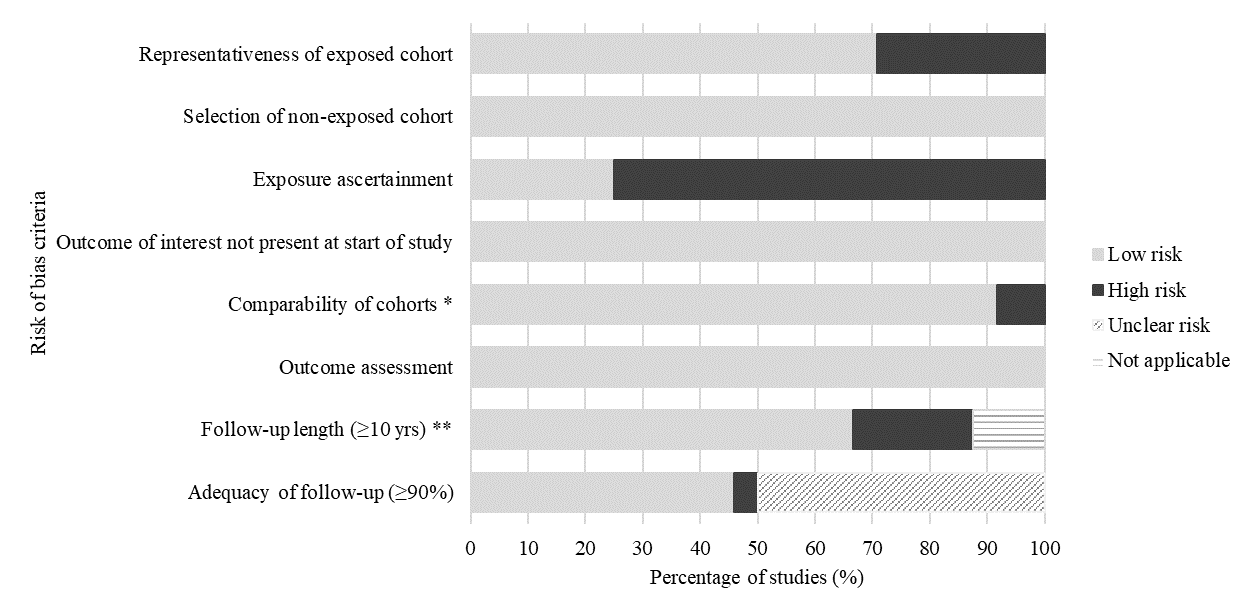


* Low risk = controlled for age, sex (where relevant), important confounders (e.g. BMI) and other relevant confounders; High risk = adjustment for age only

** at least 10 years of follow-up possible from start of study
